# Global RNA profiles show target selectivity and physiological effects of peptide-delivered antisense antibiotics

**DOI:** 10.1093/nar/gkab242

**Published:** 2021-04-13

**Authors:** Linda Popella, Jakob Jung, Kristina Popova, Svetlana Ðurica-Mitić, Lars Barquist, Jörg Vogel

**Affiliations:** Institute of Molecular Infection Biology (IMIB), University of Würzburg, D-97080 Würzburg, Germany; Institute of Molecular Infection Biology (IMIB), University of Würzburg, D-97080 Würzburg, Germany; Institute of Molecular Infection Biology (IMIB), University of Würzburg, D-97080 Würzburg, Germany; Institute of Molecular Infection Biology (IMIB), University of Würzburg, D-97080 Würzburg, Germany; Helmholtz Institute for RNA-based Infection Research (HIRI), Helmholtz Centre for Infection Research (HZI), D-97080 Würzburg, Germany; Faculty of Medicine, University of Würzburg, D-97080 Würzburg, Germany; Institute of Molecular Infection Biology (IMIB), University of Würzburg, D-97080 Würzburg, Germany; Helmholtz Institute for RNA-based Infection Research (HIRI), Helmholtz Centre for Infection Research (HZI), D-97080 Würzburg, Germany; Faculty of Medicine, University of Würzburg, D-97080 Würzburg, Germany

## Abstract

Antisense peptide nucleic acids (PNAs) inhibiting mRNAs of essential genes provide a straight-forward way to repurpose our knowledge of bacterial regulatory RNAs for development of programmable species-specific antibiotics. While there is ample proof of PNA efficacy, their target selectivity and impact on bacterial physiology are poorly understood. Moreover, while antibacterial PNAs are typically designed to block mRNA translation, effects on target mRNA levels are not well-investigated. Here, we pioneer the use of global RNA-seq analysis to decipher PNA activity in a transcriptome-wide manner. We find that PNA-based antisense oligomer conjugates robustly decrease mRNA levels of the widely-used target gene, *acpP*, in *Salmonella enterica*, with limited off-target effects. Systematic analysis of several different PNA-carrier peptides attached not only shows different bactericidal efficiency, but also activation of stress pathways. In particular, KFF-, RXR- and Tat-PNA conjugates especially induce the PhoP/Q response, whereas the latter two additionally trigger several distinct pathways. We show that constitutive activation of the PhoP/Q response can lead to Tat-PNA resistance, illustrating the utility of RNA-seq for understanding PNA antibacterial activity. In sum, our study establishes an experimental framework for the design and assessment of PNA antimicrobials in the long-term quest to use these for precision editing of microbiota.

## INTRODUCTION

Antisense oligomers, especially antisense peptide nucleic acid (PNA), programmed to target mRNAs of essential bacterial genes are an attractive technology for the design of species-specific antibiotics (1–3). Proof-of concept for antisense oligomer antimicrobials has been demonstrated with Gram-negative *Acinetobacter*, *Brucella*, *Burkholderia*, *Campylobacter*, *Escherichia, Haemophilus*, *Klebsiella*, *Pseudomonas*, *Shigella* and *Salmonella*, with Gram-positive *Clostridium, Enterococcus*, *Listeria*, *Staphylococcus*, *Streptococcus*, as well as with a *Mycobacterium* species (2,4–6). Antisense oligomers have shown potency in mouse models of sepsis and lung diseases (5,7–9). Finally, it has recently been demonstrated that antisense oligomers can also be used to manipulate resistance to conventional antibiotics by transcriptome-driven design of synergistic molecules (10–12).

**Figure 1.**
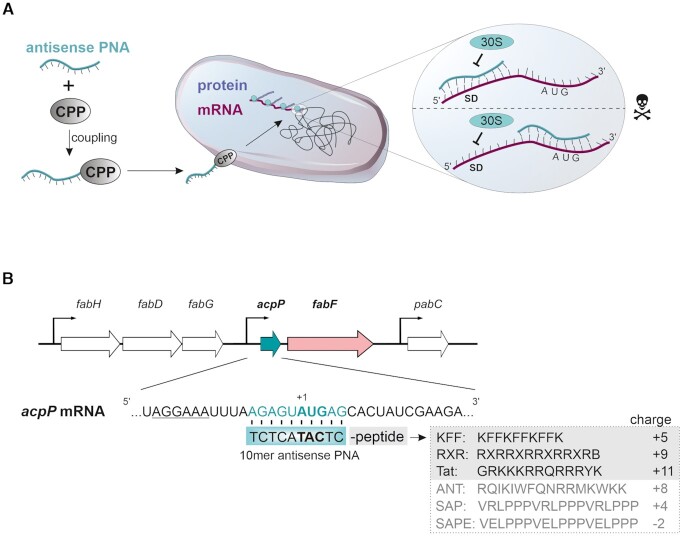
(**A**) Schematic overview of the herein applied strategy. Antisense peptide nucleic acid (PNA)-coupling to cell penetrating peptides (CPP) facilitates its delivery into the bacterial cell, e.g. *Salmonella*. PNA sequences targeting either the Shine-Dalgarno (SD) or the translational start codon (AUG) possess the strongest inhibitory efficacy in preventing ribosomal binding (30 S subunit) and thus translation. Strong bactericidal effects are elicited when the PNA is targeted against an essential gene. (**B**) Structure of the *acpP-fabF* transcription unit including a snapshot of the *acpP* mRNA sequence in *Salmonella*. The sequence of the herein used antisense PNA as well as its target sequence in *acpP* is shaded in cyan. The translational start codon is highlighted in bold type, the Shine-Dalgarno sequence is underlined. The sequence and charge (https://pepcalc.com/) of the three most potent peptides used in the present study—KFF, RXR and Tat—as well as of the additionally tested peptides—ANT, SAP and SAPE—are highlighted in gray. The indicated peptide charges correspond to peptide conjugated to PNA.

To date, most bacterial PNA studies have been end point-driven, primarily determining minimal inhibitory concentration (MIC) to assess antimicrobial activity. However, for antimicrobial PNA technology to reach its true potential, fundamental questions as to target selectivity and overall effects on bacterial cells remain to be answered. For example, with the exception of RNA external guide sequences (EGS) technology (13), the antisense oligomer is typically designed to sequester the ribosome binding site (RBS) of the target mRNA of interest, assuming translational inhibition as the primary mode of action (Figure 1A). Whether the blocked protein synthesis also affects target mRNA levels is largely unknown, but would be a prerequisite for global assessment of PNA specificity, off-target effects and efficiency by state-of-the-art transcriptomics such as RNA-seq (14).

Regarding delivery, efficient PNA uptake by bacteria requires coupling to a short (<30aa) carrier peptide (Figure 1A). Of the various cationic and amphiphilic cell-penetrating peptides (CPPs) tested thus far, different peptides penetrate different bacteria with different efficiencies and some peptides work better in Gram-positive than Gram-negative species (1,4,8,9,15). However, our mechanistic understanding of CPP-mediated entry in bacteria is poor, and information about if and how PNA conjugates trigger cellular pathways that guard the bacterial envelope is lacking completely. Thus, to design the best carrier for a target species of interest, a better knowledge of the physiological effects of PNA conjugates is needed.

The present work pioneers a systematic study of antisense PNA specificity and influence via global transcriptomics. We chose to study PNA, which is a popular antimicrobial antisense oligomer modality resistant to both nuclease and protease (16). As a model organism we chose *Salmonella enterica* serovar Typhimurium (henceforth *Salmonella*), taking advantage of the vast RNA biology knowledge and reference transcriptomes available for this important human pathogen (17–19). Adopting the essential *acpP* gene as a well-studied PNA target from *E. coli* (20), we establish that anti-*acpP* PNA delivered to *Salmonella* not only inhibits protein synthesis but also triggers rapid target mRNA decay. This allows for the use of RNA-seq to determine in a transcriptome-wide manner both the target specificity of PNA conjugates and the general physiological effects of the carrier peptides. Intriguingly, while none of three shortlisted peptides in their naked form exhibited antimicrobial effects against *Salmonella*, they induced various *acpP-*independent stress pathways for antimicrobial peptide resistance and envelope homeostasis. Our data provide insights into the selectivity of PNA-based antimicrobials and could inform rational design strategies for antimicrobial antisense oligomers in other species.

## MATERIALS AND METHODS

### Bacterial strains and peptide nucleic acids (PNAs)

The following *S. enterica* serovar Typhimurium strains were used in this study: SL1344 wt (provided by D. Bumann, MPIIB Berlin, Germany (21); internal strain number JVS-1574), SL1344 Δ*phoP* (JVS-11431), LT2 wt (provided by J. Casadesus, University of Seville, Spain; JVS-5659), LT2 PhoP^C^ (provided by S. Miller, University of Washington, Seattle, (22); JVS-8769), SL1344 Δ*hfq* (23); JVS-00584), SL1344 *rne-*701 ((24); CMA508, JVS-0870) and SL1344 *rne*R169K (chloramphenicol^R^, (25); JVS-10999). All strains were cultured in non-cation adjusted Mueller-Hinton Broth (MHB, BD Difco™, Thermo Fisher Scientific), supplemented with the appropriate antibiotic when necessary, with aeration at 37°C and 220 rpm shaking.

PNAs, peptides and peptide-conjugated PNAs (PPNAs) were obtained from Peps4LS GmbH. Quality and purity of these constructs was verified by mass spectrometry and HPLC (see Supplementary Table S1). PNAs, peptides and PPNAs (Table 1) were dissolved in water and heated at 55°C for 5 min before determining the concentration by using a NanoDrop spectrophotometer (*A*_260 nm_ for PNA and PPNA, A_205 nm_ for peptides). Aliquots of PNAs and peptides were stored at –20°C and heated at 55°C for 5 min before preparing the respective working dilutions. Low binding pipette tips and Eppendorf tubes (Sarstedt) were used throughout.

**Table 1.** Minimum inhibitory concentrations of PPNA, PNA or peptide tested against wild-type (wt) *Salmonella enterica* serovar Typhimurium strain SL1344 (∼10^5^ cfu/ml). Conjugates showing an inhibitory effect in the herein performed growth kinetics are highlighted in cyan. Growth curves for KFF-, RXR- and Tat-coupled constructs are shown in Supplementary Figures S4 and S5.

Name	Peptide sequence	PNA sequence	MIC in μM	MIC in μg/ml
KFF-*acpP*	KFFKFFKFFK	ctcatactct	1.25	5
KFF-*acpP*-scr	KFFKFFKFFK	tcactatctc	>10	
KFF	KFFKFFKFFK	-	>10	
RXR-*acpP*	RXRRXRRXRRXRXB	ctcatactct	2.5	11
RXR-*acpP*-scr	RXRRXRRXRRXRXB	tcactatctc	>10	
RXR	RXRRXRRXRRXRX	-	>10	
Tat-*acpP*	GRKKKRRQRRRYK	ctcatactct	5	22
Tat-*acpP*-scr	GRKKKRRQRRRYK	tcactatctc	>20	
Tat	GRKKKRRQRRRYK	-	>20	
ANT-*acpP*	RQIKIWFQNRRMKWKK	ctcatactct	3.3	12
ANT-*acpP*-scr	RQIKIWFQNRRMKWKK	tcactatctc	>10	
ANT	RQIKIWFQNRRMKWKK	-	0.7	1.5
SAP-*acpP*	VRLPPPVRLPPPVRLPPP	ctcatactct	>10	
SAP-*acpP*-scr	VRLPPPVRLPPPVRLPPP	tcactatctc	>10	
SAP	VRLPPPVRLPPPVRLPPP	-	>10	
SAPE-*acpP*	VELPPPVELPPPVELPPP	ctcatactct	>10	
SAPE-*acpP*-scr	VELPPPVELPPPVELPPP	tcactatctc	>10	
SAPE	VELPPPVELPPPVELPPP	-	>10	
*acpP*	-	ctcatactct	>20	

X = 6-aminohexanoic acid

B = β-alanine

Peptide and PNA sequences are represented in N to C termini orientation.

### Minimum inhibitory concentration (MIC) Determination

MIC values were determined by broth microdilution according to standard protocols with a few modifications (26). An overnight bacterial cell culture, supplemented with the appropriate antibiotic when needed, was diluted 1:100 in fresh MHB and grown to OD_600_ 0.5. The obtained culture was diluted to ∼10^5^ cfu/ml or 10^6^ cfu/ml, as indicated, in non-cation-adjusted MHB. Subsequently, 190 μl bacterial solution was dispensed into a 96-well plate (Thermo Fisher Scientific) along with 10 μl of a 20x PPNA, PNA or peptide working solution. Growth was monitored by measuring the OD at 600 nm every 20 min in a Synergy H1 plate reader (Biotek) with continuous double-orbital shaking (237 cpm) at 37°C for 24 h. The MIC was determined as the lowest concentration, which inhibited visible growth in the wells (OD_600 nm_ < 0.1).

### Determination of bactericidal effects

An overnight bacterial cell culture was diluted 1:100 in fresh MHB and grown to OD_600_ 0.5. The obtained culture was diluted to approximately 10^6^ cfu/ml in non-cation-adjusted MHB. Afterwards, aliquots of 10 μl were directly used as input condition for cfu determination and spot assay on LB agar plates. Regarding the latter, serial dilutions were prepared as indicated, to evaluate the alteration of living bacteria upon exposure to the different PPNAs. At the same time, 190 μl of this bacterial solution was placed into 2 ml eppendorf tubes along with 10 μl of a 20× on-target or scrambled PPNA solution, adjusting the indicated MICs. At the indicated time points post treatment, aliquots were used for cfu determination and spot assay.

### RNA isolation

For identification of the optimal RNA isolation method in the present study, 10^9^ cfu of Salmonella (OD_600_ = 0.5) were collected by centrifugation at 4°C and 21 100 *×g* for 5 min, and subjected to different RNA extraction protocols as described below (Figure 4A and B).

Protocol #1: RNA was extracted from bacterial pellets using RNeasy Mini kit (Qiagen) according to the manufacturer's protocol with minor modifications. In brief, bacterial cells were treated with TE buffer (pH 8.0) supplemented with 15 mg/ml lysozyme (≥35 000 U/mg, Roth) and proteinase K (>600 U/ml, Thermo Scientific). After mixing and an incubation for 10 min on ice, 3.5 volumes of RLT buffer was added, followed by addition of ethanol to adjust a final concentration of 35%. After loading the sample, columns were washed according to the manufacturer's instructions.

Protocol #2: RNA was isolated using miRNeasy Mini kit (Qiagen) with some modifications. After treating the cells with lysozyme and proteinase K as described above (protocol #1), 3.5 volumes of RLT buffer was added, followed by addition of ethanol to adjust a final concentration of 60%. After sample loading, column wash-steps were performed according to the manual.

Protocol #3: Extraction of RNA was performed by initial incubation of the cells with TE buffer (pH 8.0) supplemented with 0.5 mg/ml lysozyme at RT for 5 min, with vortexing three times each 5 s in between. Subsequently, the miRNeasy Mini kit was used and 3.5 volumes of RLT buffer was added, followed by addition of ethanol to adjust a final concentration of 60%. After loading the sample volume, columns were washed according to the manual.

Protocol #4: Isolation of RNA was performed by initial extraction using the RNA*snap™* protocol published by Stead *et al.* (27). Briefly, cells were incubated in extraction buffer (0.025% SDS, 18 mM EDTA, 1% β-mercaptoethanol, 95% formamide) for 7 min at 95°C, followed by clearance of the suspension by centrifugation at 21 100 × *g* for 5 min. Supernatant was then mixed with ethanol to adjust a final concentration of 60%. This was followed by loading the samples on miRNeasy Mini kit columns. Afterwards, washing steps were performed as described in the manufacturer's instructions.

Protocol #5: RNA was extracted using the RNA*snap™* protocol, which was followed by sodium acetate/ethanol precipitation described by Stead *et al.* (27).

Protocol #6: RNA was isolated using the Phenol (Roth)-based method. To this end, pellets were resuspended in 0.5 mg/ml lysozyme mix (in TE buffer, pH 8.0), followed by addition of sodium dodecyl sulfate (SDS) to adjust a final concentration of 1%. After inverting the tube, samples were heated at 64°C for 1–2 min and 1/10 volume of 3 M sodium acetate (pH 5.2) was added. Samples were then mixed by inversion, followed by the addition of low pH phenol. Samples were mixed and incubated at 64°C for 6 min with inverting steps in between. After centrifugation of chilled samples at 4°C, chloroform (Roth) was added to the upper aqueous phase. Samples were then centrifuged and RNA was precipitated using sodium acetate/ethanol. Briefly, 2 volumes of an ethanol:3M sodium acetate (pH 6.5) mix (30:1) was added prior to precipitation at –80°C. After washing the RNA pellet with ice-cold 75% ethanol, air-dryed RNA pellet was reconstituted in water.

Protocol #7: Extraction of RNA was performed using the TRIzol (Invitrogen)-based method according to the manufacturer's recommendations. RNA was precipitated using sodium acetate/ethanol as described above.

For genomic DNA removal, samples were subsequently treated with 0.2 U of DNase I (Fermentas) and 0.5 U Superase-In RNase inhibitor (Ambion) per 1 μg of RNA at 37°C for 45 min. Subsequently, RNA was recovered by phenol–chloroform extraction and sodium acetate/ethanol precipitation. RNA was reconstituted in water and concentration was determined using a Nanodrop spectrophotometer.

For RNA isolation post (P)PNA or peptide treatment, 2–5 × 10^6^ cfu were treated as described in ‘PNA treatment for northern blot and RNA-seq analyses’ and subjected to protocol #3 or protocol #4, as indicated.

### Rifampicin treatment for RT-qPCR analysis of *acpP* and *fabF* mRNA half-life

An overnight bacterial cell culture was diluted 1:100 in fresh MHB and grown to OD_600_ 0.5. Cells were treated with 0.5 mg/ml rifampicin and harvested at 1, 3, 5, 10, 15 and 20 min post treatment, while an aliquot prior to rifampicin treatment served as input control. RNA was isolated using protocol #1 and RNA concentration was verified using a NanoDrop spectrophotometer. After diluting RNA to an equal concentration in all samples, RT-qPCR was performed using the Takyon No ROX SYBR MasterMix dTTP Blue and Takyon One Step Converter (Eurogentec), and the appropriate primers (Supplementary Table S2) according to manufacturer's instructions. Levels of *acpP* and *fabF* mRNA were quantified using a C1000 Touch™ Thermal Cycler CFX96 Real-Time System (Bio-Rad) with the following settings: 48°C – 30 min, 95°C – 10 min, 95°C – 15 s/60°C – 1 min (40×), 21°C – ∞. The mRNA levels were normalized to the levels of 5S ribosomal RNA.

### RNA gel electrophoresis and northern blotting

Equal amounts of RNA samples (1 μg in Figure 4A; 60–100 ng in Figure 4C and D) were separated via 6% denaturing PAGE in 1× TBE and 7 M urea. Gels were stained using ethidium bromide (Roth, Figure 4A) or SybrGold (life Technologies, Figure 4C and D).

For northern blotting, equal RNA amounts were used for gel electrophoresis (10 μg in Figure 4B; 100–150 ng in Figure 4E) and subsequent transfer to Hybond+ membranes (GE Healthcare Life Sciences). After crosslinking (0,12 J/cm^2^), membranes were blocked using ROTI^®^Hybri-Quick (Roth) and incubated with RNA-specific digoxigenin (DIG; Roche)- or radioactively-labeled (5′ ^32^P-labeling (28)) oligonucleotides. *In vitro* transcription (MEGAscript T7 polymerase, Ambion), DIG-labeling of probes and northern blot detection was performed according to the manufacturer's instructions. Sequences of all the oligonucleotides (purchased from Eurofins Genomics or Sigma) employed for northern blot probe generation or RT-qPCR are listed in Supplementary Table S2. Signals were visualized on a phosphorimager (Typhoon FLA 7000, GE Healthcare) for radioactive probes, or using an ImageQuant (LAS 4000, GE Healthcare) for DIG-labeled probes.

### PNA treatment for northern blot and RNA-seq analyses

Bacterial overnight cultures were diluted 1:100 in fresh MHB and grown to OD_600_ 0.5. The obtained cultures were diluted to ∼10^6^ cfu/ml in non-cation-adjusted MHB. Afterward, the bacterial solution was transferred into low-binding 5 ml tubes (LABsolute) along with the 20× PNA, peptide or PPNA solution. The final concentrations were 5 μM for KFF- and RXR-PNAs, and 10 μM for Tat-PNAs. Equimolar concentrations were used for the respective peptide control conditions. As a negative control, cells were treated with the respective volume of sterile nuclease-free water, which was used as solvent for the test compounds.

A time kinetic was performed for subsequent northern blot analysis (5, 10, 15 min), in which the reaction was stopped at the indicated time points by using RNAprotect Bacteria (Qiagen). For RNA-seq analyses, adding RNAprotect Bacteria at 15 min post treatment stopped the reactions with the indicated PPNA constructs or the respective peptide. After vortexing for 5 s, the samples were incubated at room temperature for 10 min, followed by cell pelleting at 4°C and 21 100 ×*g* for 20 min. Pellets were directly used for RNA isolation or stored at –20°C (<2 days) until further processing. RNA was purified from bacterial pellets using isolation protocol #3, as described above (0.5 mg/ml lysozyme, miRNeasy).

### RNA sequencing

DNase treatment, cDNA library preparation and sequencing was conducted at Vertis (Munich, Germany). The RNA samples were first fragmented using ultrasound (4 pulses of 30 s each at 4°C). Then, an oligonucleotide adapter was ligated to the 3′ ends of the RNA molecules. First-strand cDNA synthesis was performed using M-MLV reverse transcriptase and the 3′ adapter as primer. The first-strand cDNA was purified and the 5′ Illumina TruSeq sequencing adapter was ligated to the 3′ end of the antisense cDNA. The resulting cDNA was PCR-amplified to about 10–20 ng/μl using a high fidelity DNA polymerase. The cDNA was purified using the Agencourt AMPure XP kit (Beckman Coulter Genomics) and was analyzed by capillary electrophoresis. For Illumina NextSeq sequencing, the samples were pooled in approximately equimolar amounts. The cDNA pool was sequenced on an Illumina NextSeq 500 system using 75 bp read length.

### Quantification of RNA-Seq data

Reads from the RNA-Seq experiments were trimmed, filtered and mapped against the *S. enterica* subsp. *enterica* serovar Typhimurium SL1344 reference genome and the three plasmids pSLT_SL1344, pCol1B9_SL1344 (other name is p2), and pRSF1010_SL1344 (19). BBDuk was applied to trim bases with a Phred quality score of <10, and adapters were removed simultaneously. Reads were then mapped against the reference genome using BBMap (v38.84) and then assigned genomic features, including both coding sequences and annotated sRNAs (19,29) using the featureCounts method of the Subread (2.0.1) package.

### Normalization and differential expression

All downstream analysis was performed using packages from the R/Bioconductor project. The raw read counts were imported and analyzed with edgeR (v3.30.0). Each set of PNA-peptide conjugate experiments (i.e. experiments with KFF, RXR and Tat peptide) was analyzed simultaneously. Genes with less than 14.6 counts per million (CPM) in at least three libraries were filtered. This cutoff value was calculated using 10/*L*, where *L* is the library size of the smallest sample (minimum library size) in millions.

Filtered libraries were examined for batch effects as proposed in (30), however no batch effects were identified. Raw read counts were normalized by trimmed mean of *M* values (TMM) normalization. Differential expression analysis was conducted using edgeR (31). Estimation of quasi-likelihood (QL) dispersions was then run with the glmFit function. Then contrasts between all samples versus the water controls were created as input for the glmQLTest function.

Genes with an absolute fold change >2 and an adjusted *P*-value (Benjamini–Hochberg, (32)) <0.001 were considered differentially expressed. The differentially expressed genes were visualized by volcano plots and heatmaps created using ggplot2 (v3.3.0) and the ComplexHeatmap (v2.4.2) package, respectively.

### KEGG pathway enrichment analysis

To identify KEGG pathways for each gene, the R package KEGGREST (v1.28.0) was used. Additionally, gene sets of regulons curated in (18) were added prior to the analysis. Rotation gene set testing (fry version of the roast gene set enrichment test (33)) was performed to identify enrichment of gene sets.

Gene sets assigned to >10 genes and FDR-corrected *P*-values <0.05 are shown in Figure 8, together with the median log_2_ FC of genes in the respective pathway. If a sample had >10 significantly enriched gene sets (e.g. RXR-*acpP*), only the 10 gene sets with the lowest FDR adjusted *P*-values are shown.

### PhoP/Q related analysis

PhoP/Q related as well as strongly differentially expressed transcripts (top 10 differentially expressed transcripts with the lowest FDR adjusted *P*-values per sample) in the present study were compared to expression data of the dataset published by Colgan *et al.* (34). Transcriptomic data of wild-type *Salmonella* grown in non-SPI-2 inducing conditions and SPI-2 inducing conditions which activate PhoP/Q, as well as a PhoP/Q deletion mutant in SPI-2 inducing conditions were extracted (in transcripts per million, TPM) and plotted as a heatmap for comparison with our data.

### Comparison of the transcriptional response to PPNA, PPNA-scrambled or peptide with polymyxin antibiotics

To investigate the differences in transcriptomic response to other antibiotics, previous results (35) were compared to our datasets, i.e. KFF-*acpP*, KFF-*acpP*-scrambled, KFF, RXR-*acpP*, RXR-*acpP*-scrambled, RXR, Tat-*acpP*, Tat-*acpP*-scrambled, and Tat. O’Rourke *et al.* measured the transcriptomic response of the *Escherichia coli* WO153 strain to a wide variety of antibiotics 30 min after exposure (35). The raw read counts of the published data set for colistin, polymyxin and respective control samples were downloaded and analyzed similarly to the PNA data sets. Polymyxin, colistin and the untreated control had 4, 3 and 9 biological replicates, respectively. TMM was used for normalization and the differential expression analysis was performed as described above. To compare the results of differential expression, *E. coli* genes were mapped to *Salmonella* genes using the tool PoFF from the Proteinortho package (v6.0.19).

In total, 1603 genes were found to be orthologous between the two datasets. A heatmap was created visualizing log_2_ fold changes for each differentially expressed gene in each data set. Genes with an absolute FC >2 and an FDR-corrected *P*-value <0.01 were considered differentially expressed in both datasets.

## RESULTS

### Potent peptides for delivering antisense PNA into *Salmonella*

Although antimicrobial antisense oligomers have been published for *Salmonella* (36,37), peptides for their efficient uptake have not been systematically tested in this species. Therefore, we selected six popular peptides of various origin and physicochemical properties, such as their net charges (Table 1; Figure 1B), as potential vehicles for a 10-mer PNA against the essential *acpP* gene (20). Four of these peptides (ANT, KFF, Tat and RXR) have been previously decribed to deliver antisense oligomers into bacteria (20,38–40), whereas the SAP and SAPE peptides were only known to traverse eukaryotic membranes (41) but have yet not been studied in the context of bacteria. To test their activity as PNA carrier against *Salmonella*, each peptide was conjugated to a PNA with full complementarity within the translational start site of *acpP* (*acpP*-PNA) (Figure 1B) or a control PNA with a scrambled nucleotide sequence (*acpP*-PNA-scr). The respective unconjugated peptide and PNA provided further controls.

Judging from minimal inhibitory concentration (MIC) using a broth dilution method (Table 1; 10^5^ cfu/ml input), *acpP*-PNA coupled to either KFF, RXR or Tat peptide (MIC values of 1.25, 2.5 and 5 μM, respectively; Supplementary Figures S4 and S5) was most effective at PNA delivery into *Salmonella*, yielding MIC values comparable with standard broad-spectrum antibiotics (42). At concentrations up to 10 μM, none of these three peptides showed inhibition on its own or together with the scrambled PNA, nor did PNA alone. In contrast, the ANT peptide gave inconclusive results (Table 1); it was effective together with *acpP*-PNA (MIC of 3.3 μM) and not *acpP*-PNA-scr (MIC >10 μM), but strongly inhibited *Salmonella* on its own (MIC of 0.7 μM). Conjugates SAP-*acpP* and SAPE-*acpP* did not inhibit growth, ruling them out as PNA carriers into *Salmonella*. Taken together, three peptides—KFF, RXR and Tat—qualified for further investigation as potent vehicles for PNA transport into *Salmonella* (Figure 1B).

**Figure 2.**
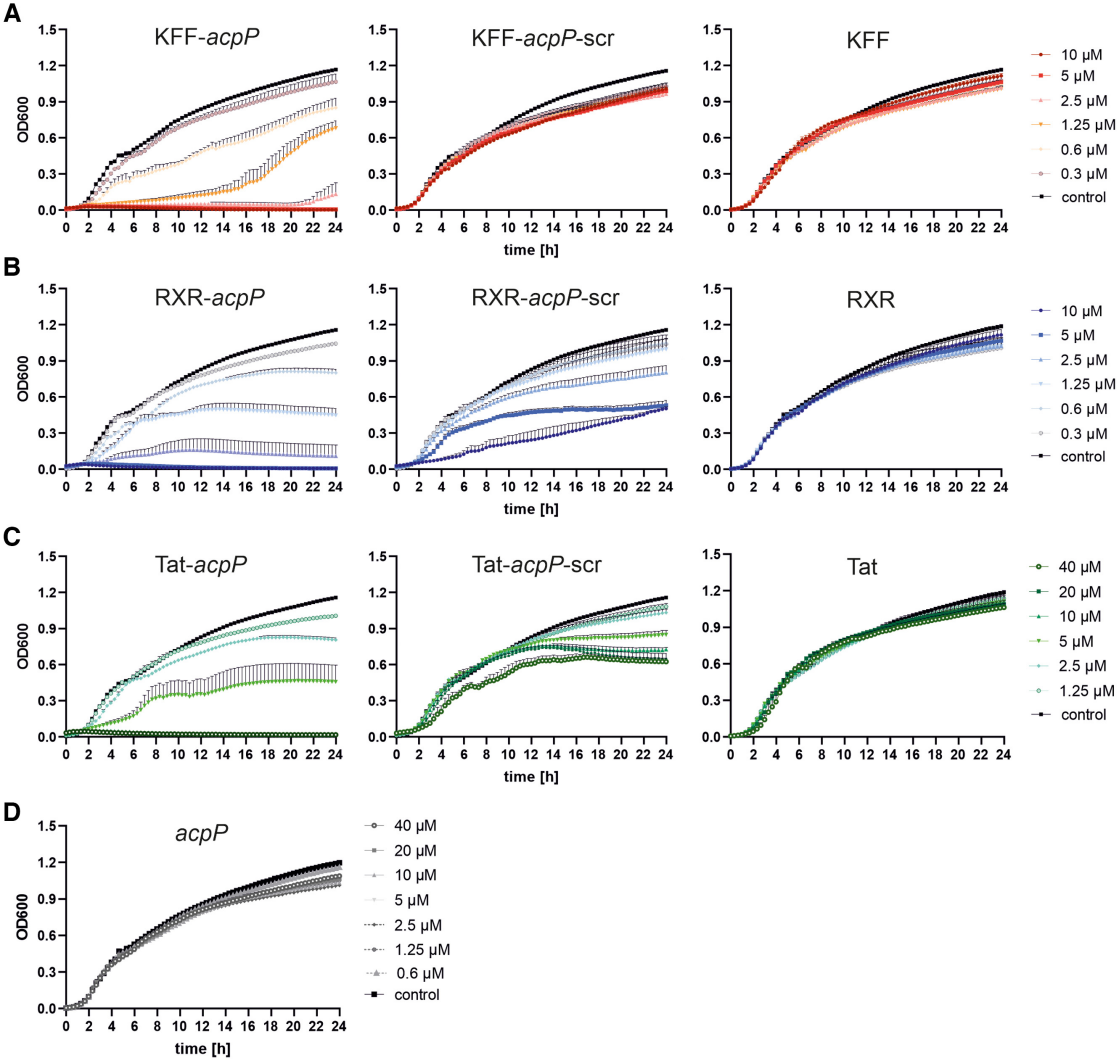
Growth kinetics and MIC determination of wild-type (wt) *Salmonella enterica* serovar Typhimurium strain SL1344 (∼10^6^ cfu/ml) in the presence of varying concentrations of the following PPNA, PNA and peptide constructs. (**A**) KFF-*acpP*, KFF-*acpP*-scrambled (scr) or KFF (10–0.3 μM), (**B**) RXR-*acpP*, RXR-*acpP*-scr or RXR (10–0.3 μM), (**C**) Tat*-acpP*, Tat-*acpP*-scr or Tat (40–1.25 μM), and (**D**) *acpP* (40–0.6 μM). Growth curves are depicted as OD_600_ (y-axis) over time (indicated in hours, x-axis). The experiment was performed three times. Error bars indicate standard error of the mean.

In order to obtain sufficient RNA for RNA-seq experiments, we further assessed the MIC of PNA-*acpP* coupled to KFF, RXR or Tat using higher bacterial input (Table 2, Figure 2; 10^6^ cfu/ml). Interestingly, when increasing bacterial input by one log, the observed MICs increased only 2–4-fold (compare Tables 1 and 2). In particular, we obtained the following MIC when subjecting 10^6^ cfu/ml to PNA treatment: KFF-*acpP* and RXR-*acpP* 5 μM, Tat-*acpP* 10 μM.

**Table 2.** Minimum inhibitory concentrations of PPNA, PNA or peptide tested against wild-type (wt) *Salmonella enterica* serovar Typhimurium strain SL1344 (∼10^6^ cfu/ml). Conjugates showing an inhibitory effect in the herein performed growth kinetics are highlighted in cyan. Respective growth curves for the indicated constructs are shown in Figure 2

Name	Peptide sequence	PNA sequence	MIC in μM	MIC in μg/ml
KFF-*acpP*	KFFKFFKFFK	ctcatactct	5	20
KFF-*acpP*-scr	KFFKFFKFFK	tcactatctc	>10	
KFF	KFFKFFKFFK	-	>10	
RXR-*acpP*	RXRRXRRXRRXRXB	ctcatactct	5	22
RXR-*acpP*-scr	RXRRXRRXRRXRXB	tcactatctc	>10	
RXR	RXRRXRRXRRXRX	-	>10	
Tat-*acpP*	GRKKKRRQRRRYK	ctcatactct	10	44
Tat-*acpP*-scr	GRKKKRRQRRRYK	tcactatctc	>40	
Tat	GRKKKRRQRRRYK	-	>40	
*acpP*	-	ctcatactct	>40	

X = 6-aminohexanoic acid

B = β-alanine

Peptide and PNA sequences are represented in N to C termini orientation.

**Figure 3.**
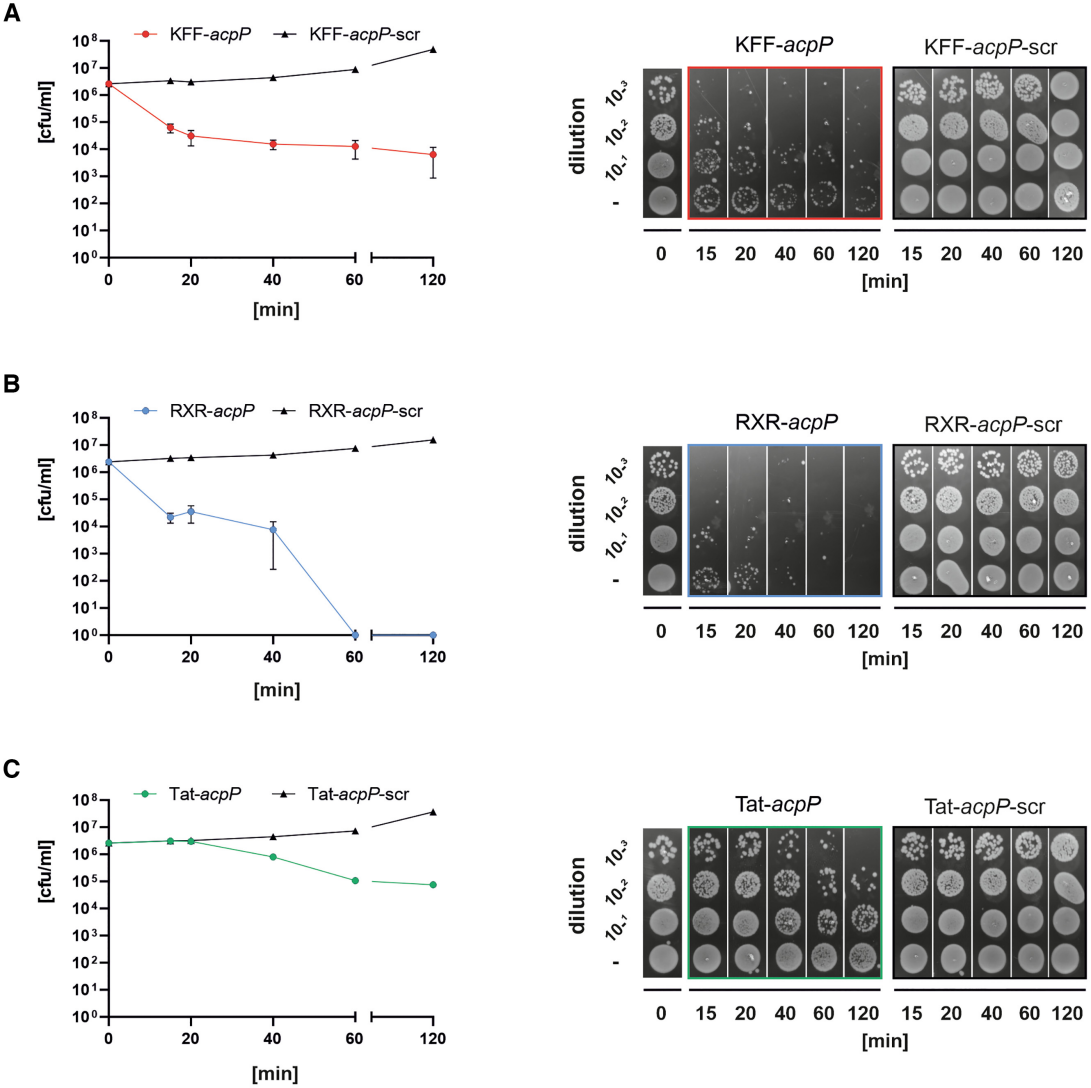
Kinetics of bactericidal effects for (**A**) 5 μM KFF-*acpP* versus 5 μM KFF-*acpP*-scr, (**B**) 5 μM RXR-*acpP* versus 5 μM RXR-*acpP*-scr and (**C**) 10 μM Tat-*acpP* versus 10 μM Tat-*acpP*-scr against *Salmonella* wt (∼10^6^ cfu/ml). After the indicated time points post treatment (15, 20, 40, 60, 120 min), aliquots of each sample were harvested to determine the number of viable cells over time (cfu/ml per min; left panels). Additional aliquots were subjected to spot assays on LB agar plates, including the indicated dilutions (right panels). The experiment was performed three times. Error bars indicate standard error of the mean.

Next, we monitored the bactericidal effects upon treatment with either of these peptide-conjugated *acpP*-PNA by applying the aforementioned MICs. After different time points post treatment, we checked for cfu/ml as parameter reflecting living bacteria (Figure 3A–C, left panels). We observed fast and strong killing upon treatment with KFF-*acpP* (A) or RXR-*acpP* (B), compared to their scrambled counterparts, which was visible from 15 min onward. In contrast to this, the bactericidal effects post Tat-*acpP* (C) treatment were time-delayed and less pronounced, showing reduced cfu/ml from 40 min onward. The killing curves were further supported by the spot assay evaluation from the respective samples (Figure 3A–C, right panels).

These combined results indicate that KFF, RXR and Tat are potent CPPs for the delivery of *acpP*-PNA into *Salmonella*, while the former two show the strongest bactericidal efficacy.

### PNA delivered into *Salmonella* induces target mRNA decay

Antimicrobial antisense oligomers, such as antisense PNAs, are typically designed to block target mRNA translation, but for RNA-seq to be able to report their activity, a selective PNA would also have to alter the steady-state level of its target mRNA, as suggested by RT-qPCR measurements in a number of previous studies (39,43–46). Moreover, translational inhibition by endogenous small RNAs (sRNAs) with similarly short antisense regions has been shown to entail rapid target mRNA decay in *E. coli* and *Salmonella* (47,48). Together, these previous findings motivated us to monitor transcriptomic changes upon short-term treatment of *Salmonella* with inhibitory concentrations of the *acpP*-PNA conjugates, as a prerequisite for the global RNA-seq analysis described further below.

**Figure 4.**
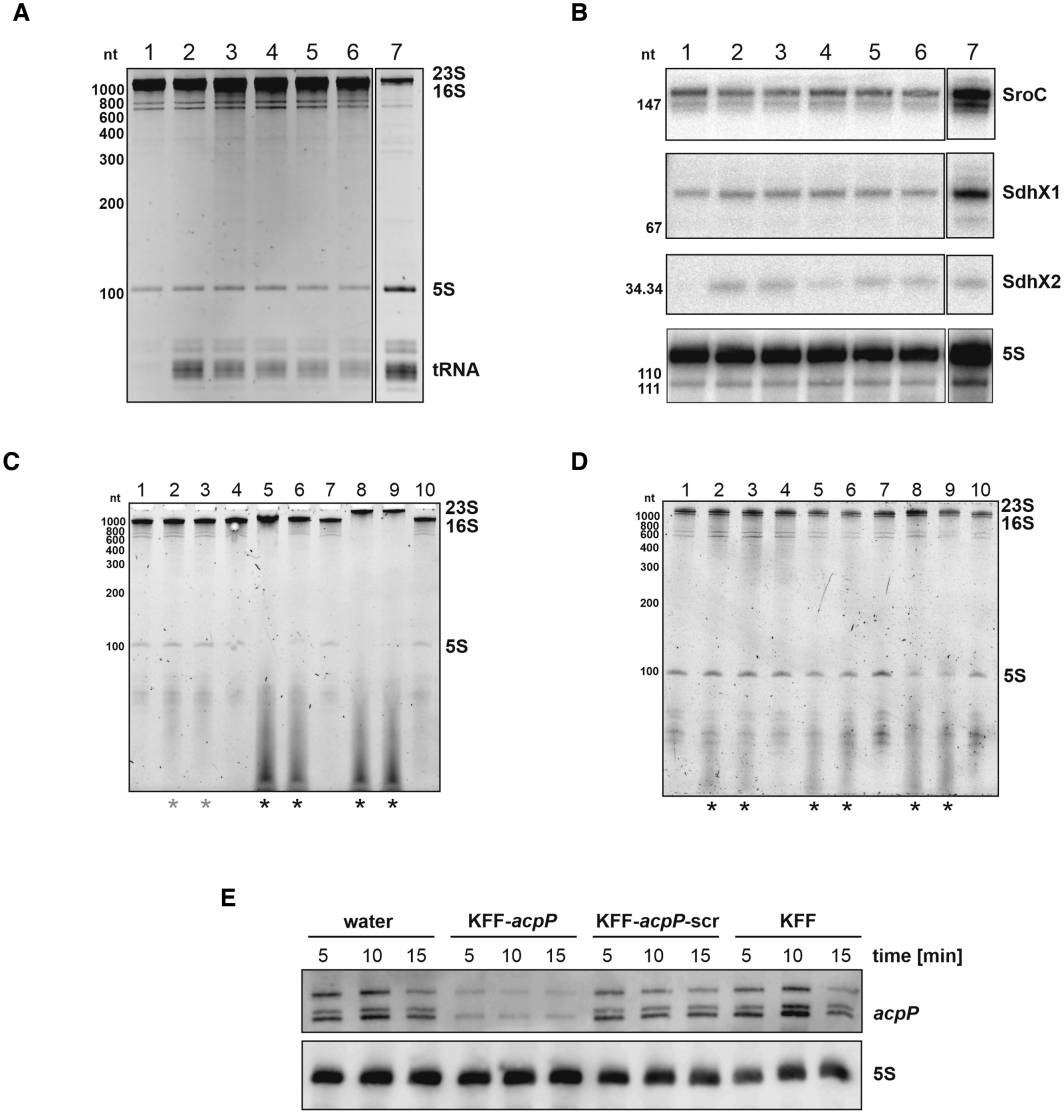
An optimal RNA isolation protocol for the analysis of PNA-mediated transcriptomic effects. (A, B) Seven different RNA isolation protocols (#1–7, described in Materials and Methods) were applied to analyze their capacity to enrich for all size classes of total RNA. In brief, 10^9^ cfu were pelleted for the isolation of RNA, which was subsequently analyzed using (**A**) a denaturing polyacrylamide gel (1 μg/lane) and (**B**) northern blotting (10 μg/lane). (A) Prominent RNAs, such as ribosomal 23S/16S or 5S, and tRNA, are indicated on the right. (B) Transcript levels of two sRNAs, SroC and SdhX (SdhX1, SdhX2), and the loading control 5S were detected using sequence specific radioactive-labeled probes. The experiment was performed two times independently. (C, D) A *Salmonella* culture (OD_600_ 0.5) was diluted to 10^6^ cfu/ml and cells were treated with the following conditions for 15 min: 1) water – untreated control, 2) 5 μM KFF-*acpP*, 3) 5 μM KFF-*acpP*-scrambled (scr), 4) 5 μM KFF peptide, 5) 5 μM RXR-*acpP*, 6) 5 μM RXR-*acpP*-scr, 7) 5 μM RXR peptide, 8) 10 μM Tat-*acpP*, 9) 10 μM Tat-*acpP*-scr or 10) 10 μM Tat peptide. After isolating RNA using (**C**) protocol #4 or (**D**) protocol #3, a total of 60–100 ng RNA was subjected to denaturing PAGE. After staining with SybrGold, ribosomal 23S/16S or 5S were visualized. Asterisks indicate the appearance of a smear in the range of short RNAs. The experiments were performed each three times independently. (**E**) A *Salmonella* culture (OD_600_ 0.5) was diluted to 10^6^ cfu/ml and cells were treated with the following constructs for 5, 10 or 15 min: water (control) or 5 μM of either KFF-*acpP*, KFF-*acpP*-scrambled or KFF peptide, as indicated. After isolating RNA using protocol #3, a total of 100–150 ng RNA was subjected to denaturing PAGE, followed by northern blotting. Transcript levels of *acpP* or the loading control *5S* were detected using sequence specific DIG-labeled probes. Results show exemplary data from one out of two biological replicates.

First, we compared different RNA isolation protocols (see Materials and Methods for detailed descriptions) to assess their capacity to enrich for all size classes in total RNA samples (10^9^ cfu in total; Figure 4A and B). While protocol #1 (lane 1) showed decreased levels of short RNAs (100–200 nt in length) in both RNA gel (Figure 4A) and northern blot analyses (Figure 4B), the TRIzol-based RNA isolation showed decreased levels of RNAs longer than 1000 nt—mirrored by weaker 23S/16S ribosomal RNA signals (Figure 4A, lane 7), but enriched levels of sRNAs (Figure 4B, lane 7). In contrast, RNA isolated using any of the protocols #2 to #6 revealed the enrichment of long as well as short RNAs (Figure 4A and B, lanes 2–6), indicating their suitability to isolate total RNA from *Salmonella*.

Second, we applied protocol #4 (Figure 4C) and protocol #3 (Figure 4D) to isolate total RNA of *Salmonella* (4–5 × 10^6^ cfu in total) upon treatment with KFF-, RXR- or Tat-conjugated PNA-*acpP*, PNA-*acpP*-scr or peptide alone. Protocol #4 is a combination of RNA*snap™* extraction (27), followed by a column-based clean up. Protocol #3 involves the same column-based clean up, but uses an initial lysozyme treatment. Both protocols resulted in the isolation of sufficient amounts of total RNA revealing mostly similar RNA patterns upon short term PNA treatment. However, there was a pronounced size shift of 23S/16S ribosomal RNA upon Tat-*acpP* and Tat-*acpP*-scr treatment when using isolation protocol #4 (Figure 4C, lanes 8 and 9), which was absent when using protocol #3 (Figure 4D, lanes 8 and 9). Furthermore, we detected a smear in the range of RNAs shorter than 100 nt upon treatment with *acpP*-PNA or its respective scrambled control conjugated to either of the three peptides (indicated by asterisks), but not post treatment with peptides alone. In summary, total RNA isolated by using protocol #3 displayed sufficient quantity and quality and was applied for our further investigations presented in this study.

Third, to test whether treatment with 5 μM on-target KFF-*acpP* results in decreased *acpP* mRNA levels in *Salmonella*, but not upon treatment with equimolar amounts of the respective scrambled control or with peptide only, we performed time kinetics for subsequent northern blotting (Figure 4E). Using an antisense probe spanning the 5′UTR and mRNA coding sequence (CDS) of *acpP*, we detected several different transcript species, similarly to earlier probing results in *E. coli* (49,50), which together with RNase E mapping results (51) we interpret to mean that processing of the *acpP-fabF* polycistronic mRNA gives rise to various stable degradation intermediates. More importantly, KFF-*acpP* treatment caused a rapid decrease of *acpP* mRNA levels from 5 min post treatment onwards, whereas *acpP* levels remained stable in the scrambled and peptide only control samples until 15 min post treatment. These decay kinetics are comparable to the results obtained upon treatment of bacteria with rifampicin (Supplementary Figure S1), according to which the half-life of both mRNAs of *acpP* and *fabF*, the second gene of the dicistron (Figure 1B) is estimated to be ∼3 min. This is in agreement with the published *acpP* mRNA half-life in *E. coli* of ∼4.4 min (52). Most importantly, the observed changes in target mRNA show that PNA treatment affects mRNA levels, encouraging us to proceed to assess three selected PPNA conjugates (Figure 1B) on a transcriptome-wide level.

### Deciphering the transcriptomic changes of *Salmonella* upon treatment with bactericidal PNA concentrations

**Figure 5.**
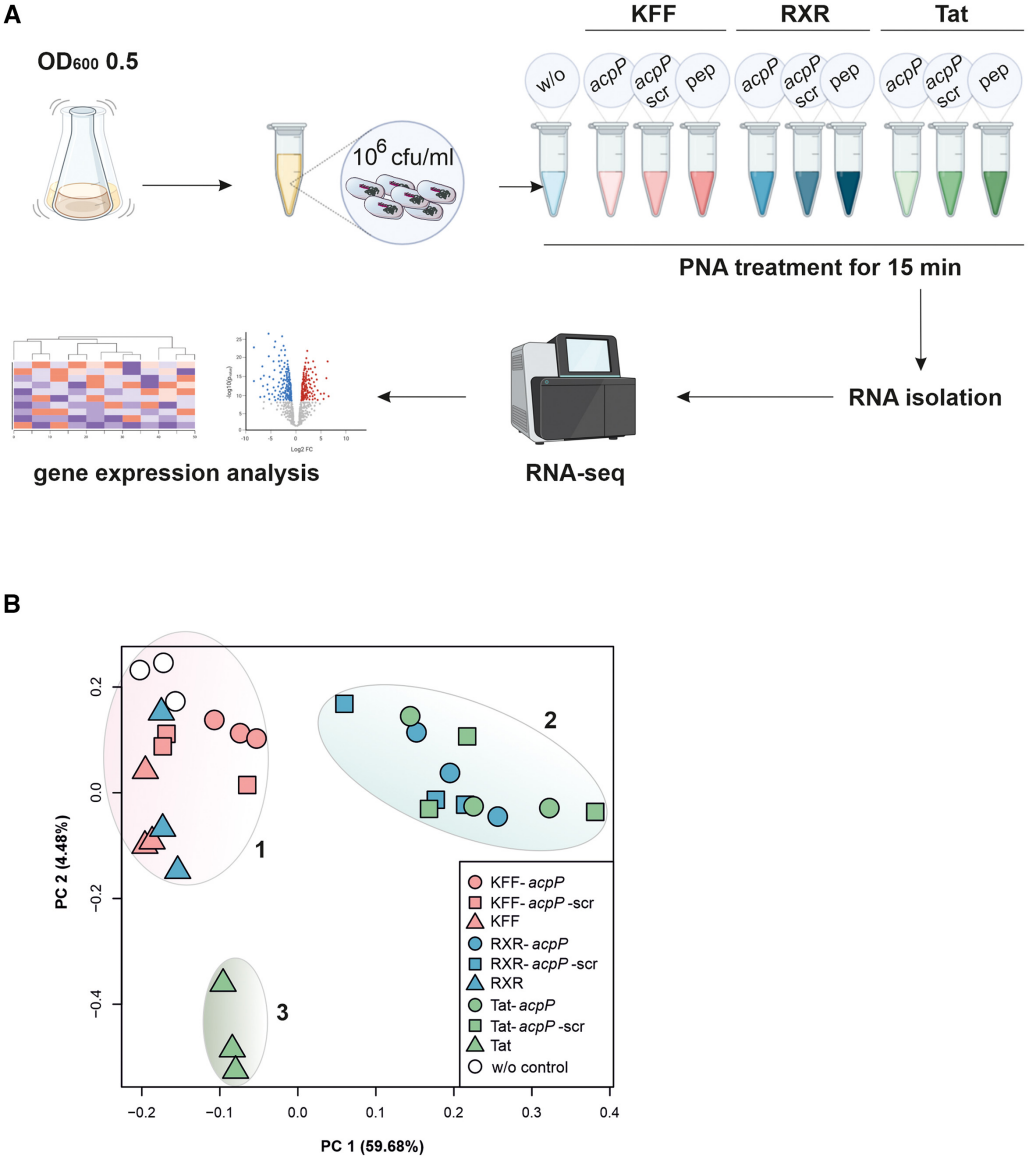
Transcriptomic profiling of *Salmonella* in response to treatment with peptide–PNA conjugates. (**A**) Experimental workflow showing the different conditions used for the analyses. ‘w/o’ denotes the untreated water control. Parts of the image have been created with BioRender.com. (**B**) Principal component analysis (PCA) of all 10 conditions including three independent biological replicates, after TMM normalization. Clusters 1, 2 and 3 were added manually after creating the plot.

Global transcriptome studies of antimicrobial responses generally use either sub-inhibitory/sub-lethal concentrations of the antibiotic of interest or analyze transcriptome profiles soon after exposure to a lethal concentration, with each approach having its pros and cons (53). Here, we opted for RNA-seq at an early time point after treating *Salmonella* with inhibitory doses of PPNAs, analyzing samples 15 min post treatment, which corresponds to approximately half a round of cell division. We obtained transcriptomes upon treatment with each of the three investigated carrier peptides (KFF, RXR, Tat), conjugated to either the (i) on-target *acpP*-PNA, (ii) the scrambled control PNA or (iii) using the peptide alone (Figure 5A).

As an initial evaluation of the dataset, we projected our data on to two dimensions using principal component analysis (PCA, Figure 5B). We found that the different treatment conditions separated into three rough clusters. In particular, all three KFF conditions (Figure 5A) and the RXR peptide treatment behaved similarly to the untreated water control (cluster 1). Cluster 2 included the treatment conditions comprising RXR and Tat conjugated either to PNA-*acpP* or PNA-*acpP*-scrambled, whereas cluster 3 was solely formed by Tat peptide treatment.

**Figure 6.**
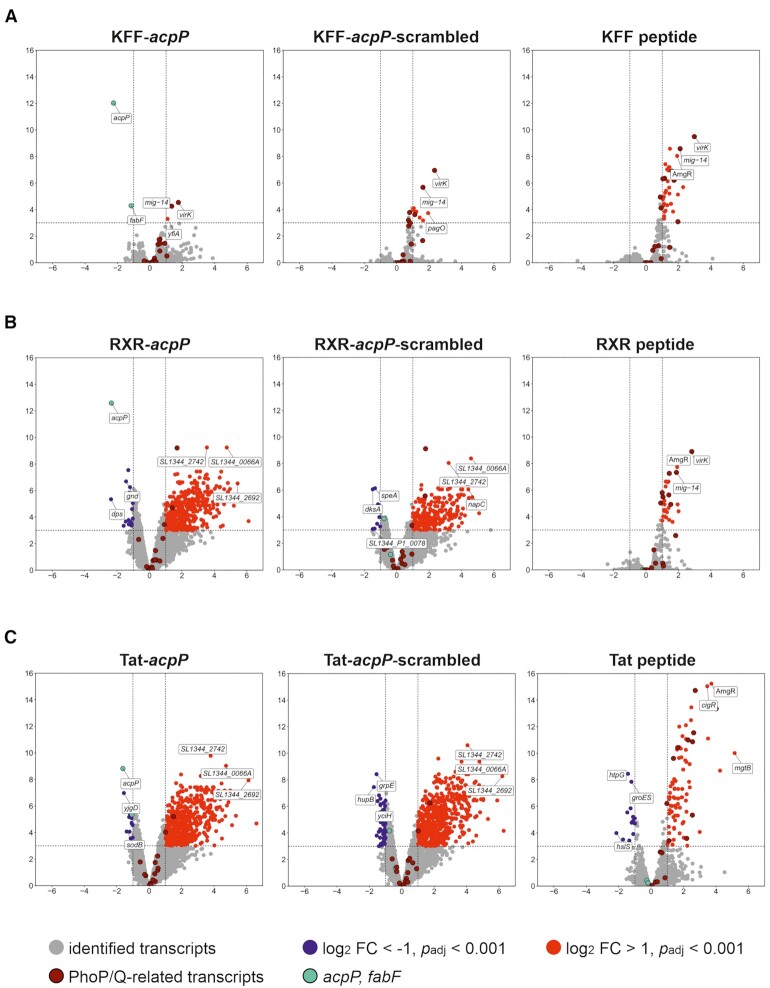
Transcriptomic responses of *Salmonella* upon PNA, PPNA or peptide treatment for 15 min. Volcano plots show calculated changes in *Salmonella* gene expression as false discovery rate (FDR)-adjusted *P*-value (–log_10_, y-axis) and fold change (log_2_, x-axis). The following conditions are shown: (**A**) KFF-*acpP*, KFF-*acpP*-scrambled or KFF peptide versus untreated (water) control, (**B**) RXR-*acpP*, RXR-*acpP*-scrambled or RXR peptide versus untreated (water) control, (**C**) Tat-*acpP*, Tat-*acpP*-scrambled or Tat peptide versus untreated (water) control. (A–C) Significantly differentially regulated genes are characterized by an absolute fold change >2 (down-regulated log_2_ < –1, up-regulated log_2_ > 1; vertical dashed line) and an FDR-adjusted *P*-value < 0.001 (–log_10_ > 3, horizontal dashed line). Significantly down-regulated genes are highlighted in blue, whereas up-regulated genes are highlighted in red. The top-3 differentially expressed transcripts, showing the strongest up- and down-regulation, are specified.

To identify transcriptomic changes that were triggered upon 15 min exposure to any of the indicated constructs in Figure 5A, gene expression levels were compared to the untreated control, while designating genes with an false discovery rate (FDR)-adjusted *P*-value < 0.001 and absolute fold change >2 as differentially expressed. The vast majority of regulated transcripts exhibited fold changes in the range of 8-fold up- or downregulation (Figure 6, Supplementary Figure S2). The broadest transcriptome responses were seen with the RXR–PNA (Figure 6B) and Tat-PNA (Figure 6C) conjugates, with 409 and 508 differentially expressed genes, respectively, most being upregulated. In contrast, RXR or Tat peptide alone triggered a much weaker transcriptomic response, reflected by the regulation of only 26 and 97 transcripts, respectively. By comparison, the KFF–PNA conjugates appeared almost physiologically silent, with only 5 and 10 differentially expressed genes in the KFF-*acpP* and the KFF-*acpP*-scr samples, respectively. However, the KFF peptide alone showed transcriptional induction of 32 genes (Figure 6A).

Importantly, the direct target of interest, *acpP*, showed selective reduction in transcript levels no matter which of the three PPNAs was used. This reduction was strongest upon treatment with KFF-*acpP* and RXR-acpP (∼4.5- to 5-fold), followed by Tat-*acpP* (∼3-fold) (Dataset S1). This degree of reduction corresponds well with the conjugates’ different MIC values (Tables 1 and 2, Figure 2). The reduction in *acpP* mRNA levels was supported by a concomitant reduction in reads from *fabF* revealing a ∼2-fold decrease post treatment with all three PPNAs. While *fabF* mRNA followed the same trend as *acpP*, its overall lesser reduction might be explained by mRNA cleavage in the *acpP*-*fabF* intergenic region, which partially uncouples the decay of the two cistrons (51,54). Attesting to the specificity of the PNA treatment, *acpP* and *fabF* mRNA levels were unaffected upon treatment with the scrambled PPNA or the peptide only controls. Together, these results establish RNA-seq as a readout for determining PNA activity *in vivo*.

### Target specific transcriptomic editing by KFF-*acpP* accompanied by the KFF-induced PhoP/Q stress response

**Figure 7.**
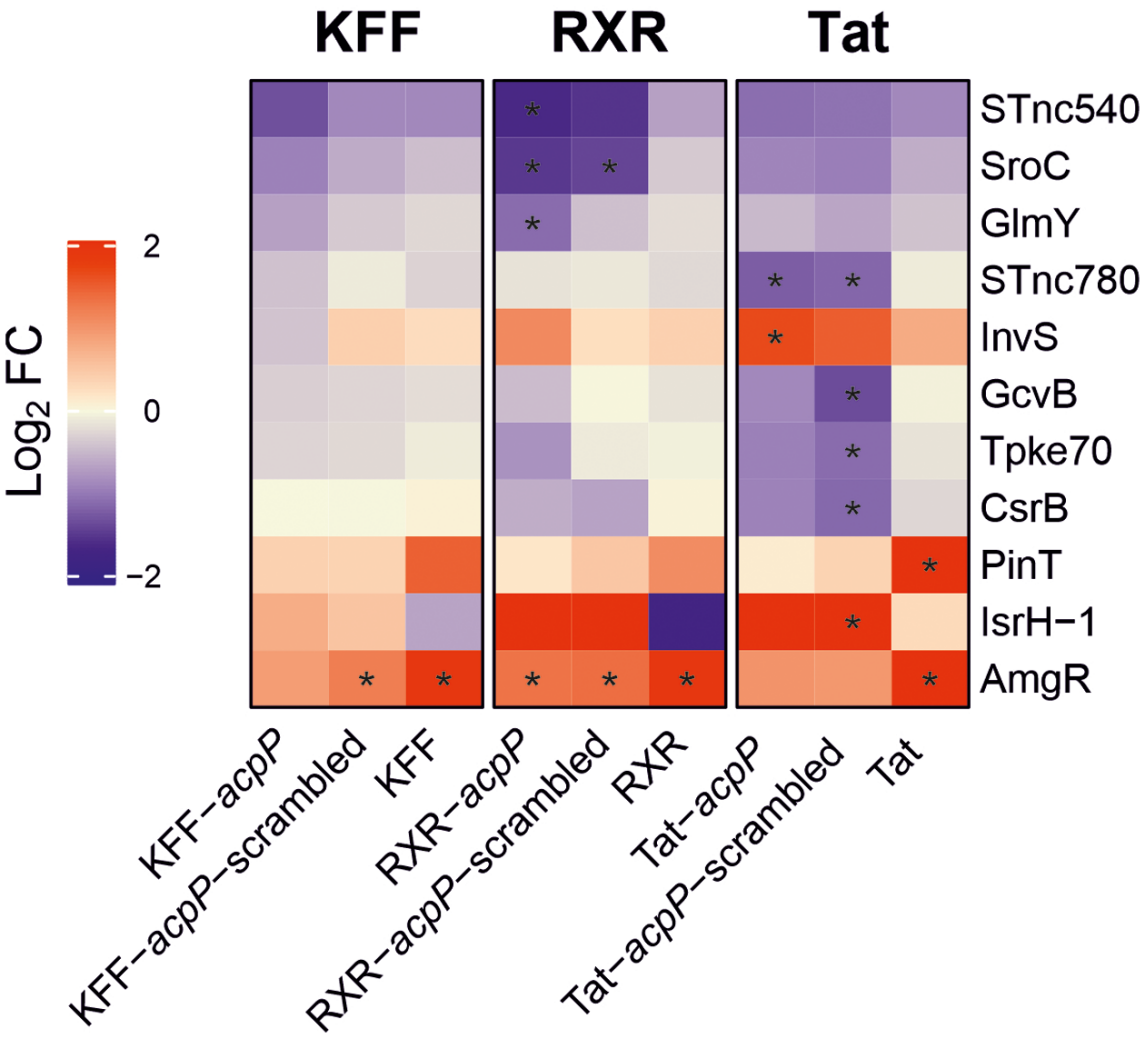
Heatmap showing differentially expressed sRNAs upon the indicated treatment conditions. Each comparison includes triplicate RNA-seq samples for the indicated conditions. The coloring indicates log_2_ fold change (FC) of the selected samples, while red and blue denote up- and down-regulation, respectively. Asterisks (*) show significantly differentially regulated sRNAs are characterized by an absolute fold change >2 (down-regulated log_2_ < –1, up-regulated log_2_ > 1; vertical dashed line) and an false discovery rate (FDR) adjusted *P*-value < 0.001.

Of all peptide-PNA conjugates tested in this study, KFF-*acpP* showed very strong bactericidal effect against *Salmonella* while at the same time having the smallest global footprint on the transcriptome (Figure 6A, Dataset S1). The three mRNAs affected in addition to *acpP* and *fabF* are *mig-14*, *virK* and *yfiA* that all show statistically significant upregulation (∼2- to 4-fold) after exposure to KFF-*acpP*. Interestingly, the inner membrane peptide sensors *mig-14* and *virK* are PhoP/Q-regulated transcripts. The PhoP/Q regulon (55,56) triggers a modulation of the protein and lipid contents of the bacterial envelope in response to, amongst other stressors, antimicrobial peptides (57,58). Treatment with KFF-*acpP*-scr and especially KFF peptide alone induced a variety of additional PhoP/Q-regulated transcripts (Figure 6A, Supplementary Figure S3, Dataset S1). Closer inspection of changes among non-coding RNAs revealed (Figure 7) that exclusively AmgR (59) is significantly upregulated upon KFF-*acpP*-scrambled (∼2-fold) or KFF treatment (∼4-fold), while induction of AmgR transcript levels upon KFF-*acpP* treatment was just below the FC threshold of 2 (FC ∼1.9-fold, *P* <0.001). Notably, AmgR is a *cis-*encoded long antisense RNA, which base pairs with *mgtC* to trigger its degradation, and is directly regulated by PhoP (59,60).

### The RXR/Tat peptide and RXR-/Tat-PNA conjugates induce large transcriptomic changes including the PhoP/Q-mediated envelope stress response

Exposure to the RXR- or Tat-PNA conjugates (both the on-target PNA and the scrambled control) caused significant changes in mRNA levels of a plethora of genes, while the respective peptides triggered a much less pronounced transcriptomic response (Figure 6B, C, Dataset S1). Intriguingly, the activated genes also included known members of the PhoP/Q regulon (55,56). In particular, the core set of upregulated PhoP/Q regulon members upon RXR-*acpP*, RXR-*acpP*-scrambled and RXR treatment were antimicrobial resistance genes *virK* and *mig-14* (RXR-*acpP* 1.89-fold, RXR-*acpP*-scrambled 1.92-fold), *pagO*, and Mg2+ transporter ATPases *mgtA*. RXR peptide alone elicited the strongest PhoP/Q response (see Supplementary Figure S3). Interestingly, *phoP* and *phoQ* themselves were also transcriptionally upregulated, though the fold change in gene expression did not exceed the imposed cutoff of >2 (*phoP* 1.99-fold, *phoQ* 1.87-fold).

With respect to treatment using Tat and Tat-PNA conjugates, the core set of induced members of PhoP/Q regulon was similar to that observed in the context of RXR (Figure 6B-C, Dataset S1), including the antimicrobial resistance genes *virK* and *mig-14*, and the membrane protein *pagO*. In contrast to RXR-PNA conjugates, the cognate Tat-PNA conjugates did not induce the Mg^2+^ transporter ATPase *mgtA*, but induced *mgtB* transcript levels, as did the Tat peptide alone. Consistent with the findings for RXR, Tat peptide alone elicited a much broader, and even stronger (maximum fold change *mgtB* 35-fold), induction of a number of PhoP/Q-regulated transcripts (Supplementary Figure S3).

**Figure 8.**
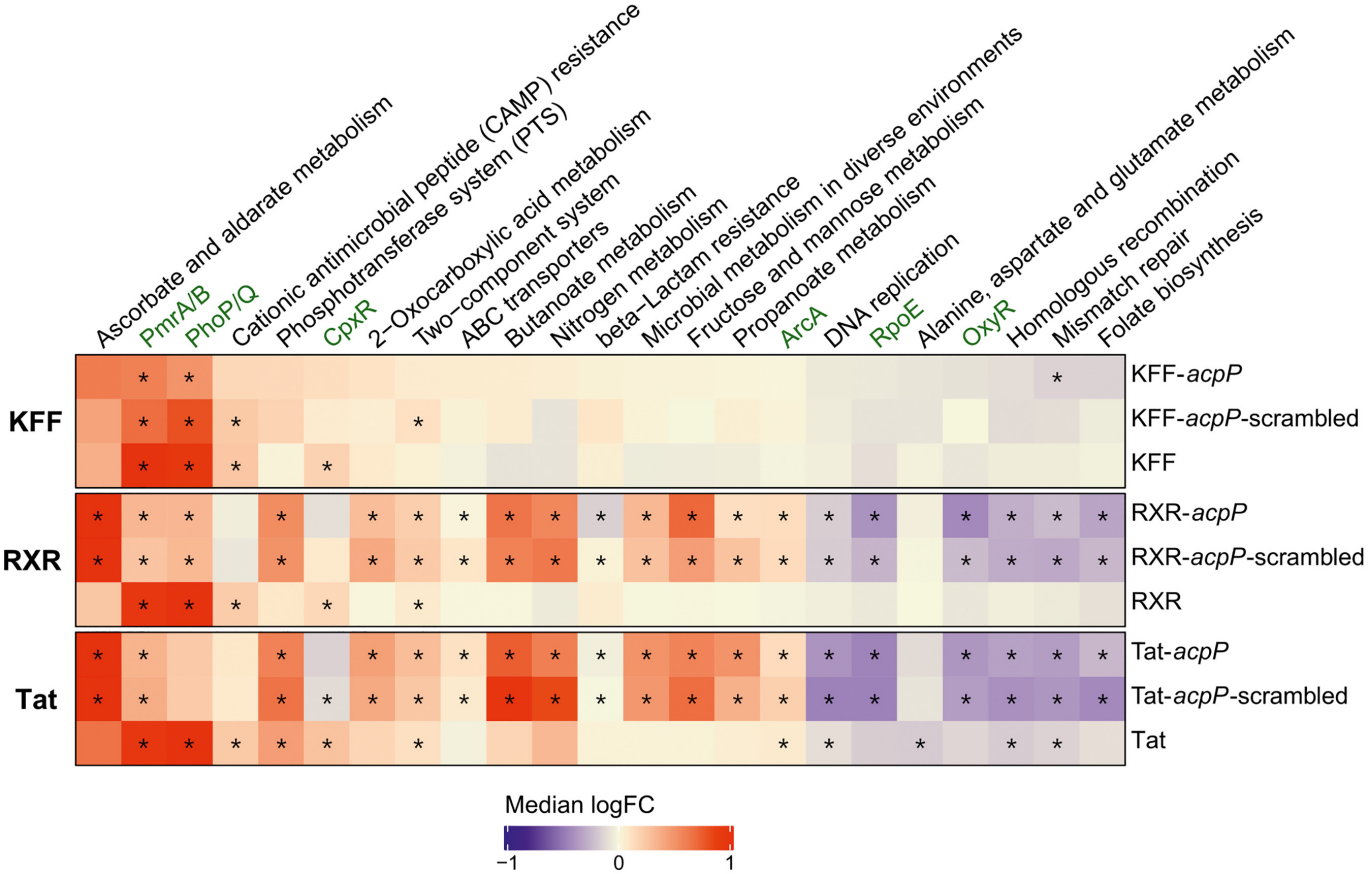
Analysis of gene enrichment classified according to annotated KEGG pathways and known regulons (marked in green, (18). Pathway and regulon analysis reveals a broad transcriptomic response upon RXR- and Tat-PNA exposure, but mostly PmrAB, PhoP/Q and CAMP-resistance-restricted gene enrichment post KFF-PNA or KFF treatment. Statistically significant (*P*-value_adj_ < 0.05) gene sets are marked with an asterisk (*). Color indicates median log_2_ fold change (FC) of all genes belonging to the gene set, while red denotes up- and blue down-regulation. Each column represents a single KEGG pathway or manually created regulon.

To put these genes into a functional context we performed a pathway enrichment analysis of differentially regulated genes using fry in the edgeR package (33) and the KEGG database (61) supplemented with regulon annotations derived from the literature (Figure 8) (18). This analysis revealed a defined response upon treatment with any of the three KFF constructs, limited to the induction of the PhoP/Q and PmrA/B pathways and genes involved in cationic antimicrobial peptide (CAMP) resistance. The PmrA/B TCS is indirectly activated by PhoP/Q and mediates LPS modifications known to increase resistance to CAMPs and polymyxin antibiotics (62). In contrast, we found several distinct pathways to be enriched upon RXR- and Tat-PNA or RXR and Tat peptide treatment, including significantly up- or downregulated transcripts. While the PhoP/Q response is significantly enriched in all three RXR-related conditions, only Tat peptide yielded significant enrichment of the PhoP/Q regulon. All tested conditions led to an enrichment in upregulation of the PmrA/B regulon, with the strongest effects seen for treatments with the peptides alone. All three peptides alone also activated the CpxR regulon, suggesting interference with protein folding in the cell envelope. Notably, the treatment with RXR- and Tat-PNA conjugates resulted in the induction of genes implicated in ascorbate and aldarate metabolism, whereas RXR and Tat peptide did not trigger this pathway.

Taken together, our data indicate that of the three tested peptides, Tat-PNA conjugates elicited the strongest transcriptomic response in *Salmonella*, suggesting that PNA-coupled Tat as well as both unconjugated and PNA-coupled RXR are recognized as potential antimicrobial peptides, similar to the toxic peptides encountered by *Salmonella* when it infects eukaryotic hosts (63). In contrast, both KFF-conjugates as well as the peptide itself trigger a very restricted set of genes, mostly belonging to the PhoP/Q stress response.

### The non-coding (small) RNA signature upon treatment with RXR- or Tat-conjugates

In *Salmonella*, non-coding RNAs can be excellent markers of the induction of a given stress response, as their promoters are often amongst the most tightly regulated ones within a given regulon (see, e.g. (18,48,64)). To narrow down the observed transcriptomic response, we restricted our differential gene expression analysis to non-coding (small) RNAs (Figure 7). In this respect, RXR and Tat peptide increased the expression of AmgR, which is consistent with the findings for KFF peptide. However, treatment with Tat peptide additionally triggered expression of the sRNA PinT, which again reflects the induction of the PhoP/Q stress response. PinT has been reported to regulate the stability or translation efficiency of a variety of *Salmonella* encoded mRNAs (18,65,66).

While the transcriptomic changes of non-coding RNA were limited to the upregulation of the aforementioned two transcripts post treatment with RXR or Tat peptide, we found additional regulated sRNAs upon RXR- or Tat-PNA treatment. In particular, our analysis revealed four and three downregulated sRNAs in the context of RXR-*acpP* and RXR-*acpP*-scrambled treatment, respectively, apart from the induction of AmgR. In both conditions, we observed a decrease in SroC and STnc1110 expression levels (Figure 7). SroC is best known as sponge of the regulatory GcvB sRNA (25,67), but it has also been implicated in mediating antibiotic resistance via regulating the levels of MgrR, and its downregulation was shown to increase the sensitivity of *Salmonella* to polymyxin B (68). In the context of Tat, we identified five and 13 sRNAs to be differentially expressed upon Tat-*acpP* and Tat-*acpP*-scrambled treatment, respectively, apart from AmgR upregulation (Figure 7). Both data sets showed the decrease of STnc780, CsrB, GcvB and ArcZ transcript levels. Notably, CsrB sRNA post-translationally regulates the protein levels of the RNA-binding protein CsrA, which modulates translation of its target mRNAs (69). The sRNA GcvB, and its homologue in *E. coli*, were shown to post-transcriptionally control the expression of ABC transporters for amino acids and peptides (70).

### PhoP/Q mediates *Salmonella* resistance to certain PPNA antibiotics

Since PhoP/Q was among the most strongly activated pathways in our analysis (Figure 8), we sought to estimate the number of PhoP/Q-regulated genes in our datasets, incorporating published gene expression data in *Salmonella* wild-type and PhoP/Q-knockout strains under SPI-2 inducing conditions that activate the PhoP/Q regulon (17,34). A number of these transcripts were up-regulated in the RXR-PNA and Tat-PNA datasets, and to a lesser extent in the KFF-PNA dataset, indicating the involvement of this system in sensing and responding to peptide-conjugated PNAs (Supplementary Figure S3).

To further elucidate the impact of PhoP/Q on PPNA activity, we determined the MICs for *Salmonella* Δ*phoP* or PhoP^C^ strains, which either lack the PhoP response regulator of this two-component system (TCS) or encode a constitutively active PhoP protein, respectively (71). The deletion of *phoP* did not alter the MIC in the context of KFF-*acpP* (Table 3, Supplementary Figure S4A), despite slightly reduced growth of the Δ*phoP* strain at one sub-MIC concentration (0.6 μM), compared to the respective wild-type *Salmonella* condition (Supplementary Figure S4A). Likewise, a constitutively active PhoP/Q response (PhoP^C^ strain) did not influence the inhibitory activity of KFF-*acpP* (Table 3, Supplementary Figure S5A). We hypothesize that while the KFF peptide triggers the PhoP/Q response, the following physiological changes and membrane remodeling do not affect KFF-mediated transit of the bacterial envelope.

**Table 3.** Minimum inhibitory concentrations of KFF-*acpP*, RXR-*acpP* and Tat-*acpP* conjugates tested against wt *Salmonella enterica* serovar Typhimurium strain SL1344 versus its isogenic mutant Δ*phoP*, and wild-type *Salmonella enterica* serovar Typhimurium strain LT2 versus its isogenic mutant PhoP^C^ (∼10^5^ cfu/ml). Cyan background denotes the conjugate for which MIC values against *Salmonella* mutants differed from those of the respective wt. Growth curves are shown in Supplementary Figures S4 and S5

	MIC in μM			
Name	SL1344 wt	SL1344 Δ*phoP*	LT2 wt	LT2 PhoP^C^
KFF-*acpP*	1.25	1.25	1.25	1.25
KFF-*acpP*-scr	>10	≥10	>10	>10
KFF	>10	10	>10	>10
RXR-*acpP*	2.5	1.25–2.5	1.25	2.5
RXR-*acpP*-scr	≥10	5	10	>10
RXR	>10	>10	>10	>10
Tat-*acpP*	5	2.5	2.5–5	10
Tat-*acpP*-scr	>20	20	>20	>20
Tat	>20	>20	>20	>20
*acpP*	>20	>20	>20	>20

The activity of the RXR-*acpP* conjugate was slightly affected by the Δ*phoP* or PhoP^C^ mutations compared to the respective wild-type background (Table 3, Supplementary Figure S4B and S5B). In particular, we observed a 2-fold increase in the MIC upon RXR-*acpP* treatment in the PhoP^C^ background compared to the wild-type strain, while the susceptibility towards RXR-*acpP* was greatly increased at one sub-MIC concentration (1.25 μM) in the Δ*phoP strain*.

In sharp contrast, we observed a clear phenotype with the Tat-*acpP* conjugate. We found that the Δ*phoP* strain, which is unable to successfully mount a PhoP/Q response, exhibited a 2-fold reduction in the MIC (2.5 μM) compared to wild-type *Salmonella* (5 μM). Reciprocally, a constitutive PhoP/Q response rendered *Salmonella* at least two to four times more resistant (MIC of PhoP^C^: 10 μM, isogenic wild-type: 2.5–5 μM) to Tat-*acpP* (Table 3, Supplementary Figures S4C and S5C). It has to be noted that our RNA-seq analyses, performed 15 min post treatment, did not show a significant enrichment of known PhoP/Q-associated transcripts in Tat-PNA treated samples. However, since the bactericidal effect of Tat-*acpP* was visible only from 40 min onward (Figure 3), it is likely that the effects on PhoP/Q-regulated transcripts would be more pronounced at later time points as seen for treatment with the Tat peptide alone.

In summary, the combined data clearly illustrate the power of RNA-seq-based transcriptomic profiling to find putative resistance factors upon PNA treatment that should be considered in antisense oligomer therapy design.

## DISCUSSION

Proof-of concept for antibacterial antisense oligomers was established decades ago (72,73), and efficacy has since been demonstrated in more than a dozen bacteria, mostly pathogens (2,3). Yet, to leverage their full potential as programmable, species-specific antibiotics, especially in complex communities, we must develop an understanding of their full direct and indirect activities upon uptake by the bacterial cell, and of the underlying principles of target recognition. This applies to antisense oligomers that directly sequester mRNAs (72) or program an endogenous nuclease for mRNA cleavage (13) alike.

Our study provides the first experimental and computational framework for the global assessment of the target specificity and physiological effects of PNA antibiotics. The RNA-seq profiles obtained in response to treatment with different peptide-PNA conjugates demonstrate that anti-*acpP* PNA delivered to *Salmonella* not only inhibits protein synthesis but also triggers rapid target mRNA decay. Moreover, while none of the carrier peptides in their naked form exhibited antimicrobial effects against *Salmonella*, peptide-PNA conjugates, especially RXR and Tat, induced various *acpP-*independent stress pathways for antimicrobial peptide resistance and envelope homeostasis. Below we will discuss the importance of having a global method such as RNA-seq available for PNA characterization, how this global data may be used to improve PNA design, and what can be learned from this data about intrinsic stress and resistance mechanisms that must be understood for PNAs to be used as precision tools in complex microbial communities.

### A global view of PNA activity and mechanistic implications

The power of large-scale transcriptomics methods such as RNA-seq lies in their potential to detect not only direct effects of a PNA on target gene levels, but also its indirect effects. In the case of antibacterial PNAs, mRNA antisense regulation has been the assumed mode-of-action, but evidence for this has primarily come from two observations obtained on individual target genes: i) antisense oligomers are active when antisense to the mRNA sequence but not the complement; ii) they work best at the RBS, i.e., the most crucial mRNA region for protein synthesis (20,74–76). Against this backdrop, our RNA-seq experiments monitoring in parallel the activity of all ∼5000 *Salmonella* genes for the first time pinpoint specific mRNA repression of the target of interest (here, *acpP*) as the primary outcome of antibacterial PNA activity. Induction of DNA repair systems, which would signal targeting of the chromosome, is not observed.

The data also allows for conclusions regarding potential off-target effects. The 10-mer antisense *acpP* PNA sequence used here has eight fully complementary binding sites within the *Salmonella* genome, with four located within the genes *pgtA, tdcC, udhA* and SL1344_3499. Inspection of these sites reveals that all of them reside within the CDS, i.e., downstream of the translation initiation site. Importantly, our RNA-seq dataset did not show differential expression of any of these additional genes upon PNA treatment.

The scrambled PNA has seven fully complementary binding sites in the genome of *Salmonella*, including two within CDSs (*sbp* and SL1344_2710), but not in proximity to the translational start codon. Of these two putative off-targets only *sbp* is differentially upregulated in the Tat-*acpP*-scr and in the RXR-*acpP*-scr samples. However, *sbp* is also significantly upregulated in the RXR-*acpP* and Tat-*acpP* samples, which have no complementarity to those transcripts. Given that none of these effects clearly segregate with scrambled control samples, we exclude sequence-related off-target effects by scrambled controls.

The complete absence of off-targeting in these aforementioned genes mirrors mechanistic conclusion from the endogenous Hfq-dependent sRNAs in *Salmonella*, which operate within a ∼50 bp window around the start codon in order to repress target mRNAs (77–79). While productive CDS targeting has also been reported for sRNAs (80,81), it has remained the exception, and seems to involve the formation of larger ribonucleoprotein complexes with Hfq and RNase E for the paired sRNA to successfully withstand displacement by elongating 70S ribosomes (82). By contrast, a 10-mer *acpP*-PNA paired to an internal mRNA region is unlikely to withstand the strong RNA helicase activity of elongating ribosomes, which can disrupt a perfect 27-bp helix with predicted melting temperature of 70°C (83). Thus, our RNA-seq results suggest that algorithms for predicting highly selective PNAs, e.g. for rewiring metabolic networks within the microbiota, should give less weight to CDS than to RBS pairing.

We do caution, however, that although an inverse correlation between ribosome occupancy and mRNA stability is well-accepted, at least for Gram-negative bacteria (84,85), we cannot conclude with certainty that mRNA downregulation upon treatment with an antisense PNA will be universal, and proteome-based methods will be required to address this question. Nonetheless, our RNA-seq framework now opens the possibility of investigating specificity, off-target effects and bacterial responses using libraries of PNA variants in multiple microorganisms. Recent technological advances in the field have already decreased the experimental costs of RNA-seq dramatically, and new sample processing methods such as RNAtag-seq for library pooling (86) and DASH for rRNA depletion (87) promise significant further cost reductions (down to $5–10 per sample).

Inasmuch as the observed rapid target mRNA decay upon PNA treatment is useful for enabling global analysis, it also raises new questions, for instance: does the PNA-mediated sequestration of the target RBS accelerate the intrinsic decay of the *acpP* mRNA, as previously observed with overexpressed Hfq-dependent sRNAs (47,48)? What ribonucleases are responsible for the cleavage and are these actively recruited? What structure—PNA-RNA duplex or the unprotected parts of mRNA—do they recognize in the PNA-treated samples?

**Figure 9.**
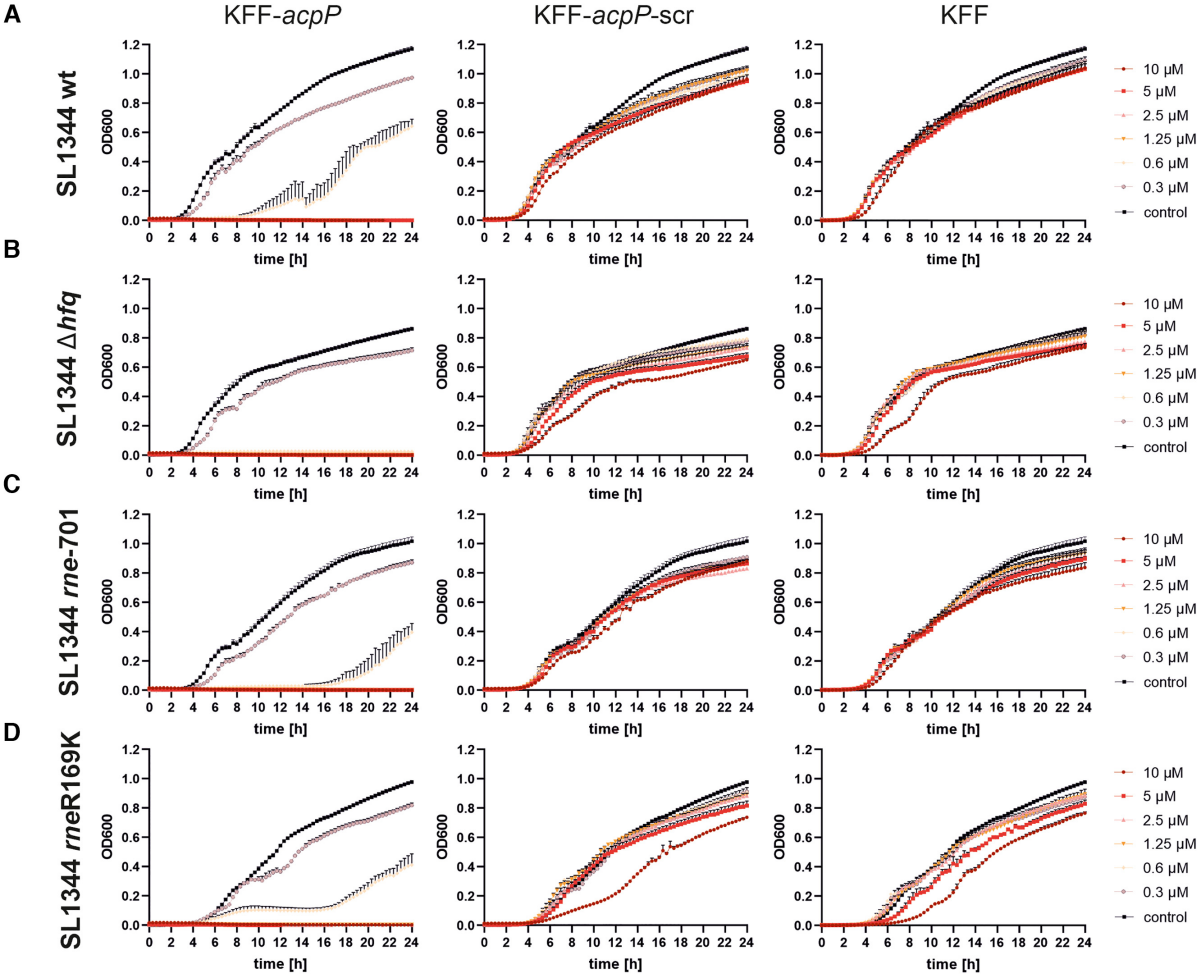
PNA-mediated growth inhibition is independent of established factors for regulation by endogenous small RNAs. Growth kinetics for MIC determination of 10^5^ cfu/ml of the indicated *Salmonella* strains (**A–D**) in the presence of varying concentrations of KFF-*acpP*, KFF-*acpP*-scrambled (scr) or KFF peptide in serial dilutions from 10 μM to 0.3 μM. Data represent the average of three biological replicates, error bars indicate standard error of the mean (SEM).

The essential RNase E, being the ribonuclease responsible for the decay of most transcripts in proteobacteria (88–90), represents an attractive target for detailed investigation of PNA-induced target decay. Our earlier study using TIER-Seq revealed a number of potential RNase E cleavage sites within the *acpP* mRNA sequence (51), eight in the gene coding region, six in the 5′ untranslated region, and one located downstream in *fabF*. Involvement of RNase E or another ribonuclease in target mRNA decay can now be examined definitively by performing similar transcriptomics studies in RNase-deficient strains of *Salmonella* (91). To elucidate whether the *acpP* PNA-inherent antibacterial activity due to target mRNA decay involves functional Hfq, RNase E degradosome or RNase E 5′ sensor activity in *Salmonella*, we performed MIC assays with the respective mutant strains (Figure 9). However, the MICs of these mutant strains did not differ from the wild-type strain, therefore it is tempting to speculate that the PNA-mediated target mRNA decay is likely different from that induced by natural sRNAs.

### Impact of PNA on bacterial physiology

Starting with pioneering work on *Mycobacterium tuberculosis* using microarrays (92), global gene expression analysis has been increasingly used to understand the cellular response to antibiotic treatment. These previously studied antibiotics work by various mechanisms, which are often reflected in altered transcript patterns. For example, the DNA damage effects of fluoroquinolones are visible by an upregulation of the bacterial SOS response (93), while exposure of *Salmonella* to sub-inhibitory concentrations of chlortetracycline and florfenicol resulted in increased expression of multiple genes involved in virulence and pathogenicity (94).

Indeed, our RNA-seq analysis identified numerous genes up-regulated in presence of Tat-*acpP* PNA, RXR-*acpP* PNA, as well as their corresponding scrambled PNA-peptide controls (Figure 3B). Many of these are targets of the two-component virulence regulation system PhoP/Q, a key player in the bacterial defense system against antimicrobial peptides encountered in the host, which was also strongly induced upon KFF-PNA or KFF peptide treatment. We note that while bacterial stress responses often show double-digit changes (17), the changes seen here are less dramatic. This observation could be either the result of 15 minutes post-exposure being a suboptimal time point to observe full PhoP/Q induction, or could be due to suboptimal activation of PhoP/Q by the applied concentrations of peptides and PNA conjugates. Maximal activation of the PhoP/Q response in our assays was seen for treatment with the three unconjugated peptides, perhaps suggesting conjugation with PNA interferes with peptide sensing by PhoQ (95).

A multitude of additional genes involved in distinct pathway was induced in both RXR-/Tat-*acpP* as well as -*acpP*-scrambled datasets, which indicates that peptide-PNA conjugates elicit broad transcriptomic responses irrespective of the attached PNA sequence. In contrast, the KFF-PNA conjugates and the KFF peptide alone, on the other hand, appeared to exclusively trigger the PhoP/Q regulated response, without greatly affecting additional pathways. According to the comparative analysis between KFF, RXR and Tat carrier peptides, the former would be the carrier of choice for *in vitro* studies analyzing the efficacy of different PNAs or distinct oligonucleotide mimics in *Salmonella*. With respect to clinical applications of PNA conjugates as anti-infectives, however, it needs to be stressed that KFF would not be a good choice due to fast resistance development via mutations in the *sbmA* gene (44,96).

Several different systems including TCS-based PhoP/Q and PmrA/B guard the bacterial envelope (97), allowing *Salmonella* to sense and respond to the presence of toxic cationic peptides in the environment. Upon peptide binding, they initiate regulatory cascades leading to restructuring of the envelope, making it less penetrable to the AMPs, as well as increasing the expression of efflux pumps and even extracellular proteases which digest AMPs before they could enter the cell. All three PNA-conjugated peptide constructs as well as the naked peptides induced expression of the inner membrane peptide sensors VirK and Mig-14. Both proteins have been proposed to directly bind AMPs (98,99), hindering their entrance into the cell. Interestingly, the upregulation of the lipid A modifying enzyme PagP upon KFF, RXR and Tat peptide treatment might mediate restructuring of the LPS layer on the outer membrane, reducing its negative net charge and thus decreasing the AMP binding to the cell surface (100). Additionally, upregulation of the transcriptional activator PmrD upon peptide treatment indicates recruitment of PmrA/B, confirmed by our enrichment analysis, which likewise is responsible for protective LPS modifications in response to AMPs (101–103).

These systems have well-characterized roles in resistance to polymyxin antibiotics, thought to be mediated primarily by changes in the charge of the LPS (104). Of the three peptides tested, Tat carries the highest net charge of +10, followed by +8 for the RXR with +4 for the KFF peptides (Figure 1B). Our experiments with the *phoP* deletion and constitutively active mutants confirm the influence of this system on *Salmonella* resistance towards the highly charged Tat-PNA (Table 3, Supplementary Figures S4 and S5), emphasizing the potential for PPNA resistance development in *Salmonella* on the basis of already existing peptide defense mechanisms. Additionally, differential expression of cytoplasmic and periplasmic chaperones (*groES/groEL htpG* and *dnaJ/dnaK)* or heat shock genes (*hslS/hslT*) indicates the development of a broader stress response upon *Salmonella* exposure to Tat peptide

To further interrogate similarities in the response to polymyxin antibiotics and PPNA, we compared our results to publicly available RNA-seq data sets for *E.coli* treated with subinhibitory concentrations of polymyxins (35). We observed that in general, gene expression changes in response to colistin and polymyxin B are significantly different (Supplementary Figure S6). In particular, KFF treatments show a completely different pattern of differentially regulated genes compared to either polymyxin treatment. The gene expression profiles in response to RXR and Tat conjugates, however, showed some overlap with the response to polymyxins. Interestingly, the peptides alone were more dissimilar to treatment with conjugates. This suggests that even if PPNAs induce some similar stresses in *Salmonella*, the cellular response to PPNA treatment is largely distinct from polymyxins.

A number of open questions remain regarding the activity of PPNA antimicrobials. To date, little is known about how PPNAs traverse bacterial membranes. It is generally assumed that in Gram-negative bacteria PPNAs passively cross the outer membrane through one of the porin systems, and only one peptide transporter SbmA has been implicated in transport of KFF-PNAs across the inner membrane (44). The *sbmA* gene is not differentially expressed in any of our datasets, which argues against the possible existence of a dedicated response system leading to shutdown of its expression under the herein tested conditions. We could, however, observe upregulation of the inner membrane protein PagO in KFF-PNA, RXR-PNA and Tat-PNA treated samples (Figure 6, Dataset S1). PagO is a putative member of the EamA transporter family, suggested to function as an amino-acid metabolite efflux pump (105). Furthermore, the gene *yohJ* encoding a putative inner transmembrane protein was significantly induced upon exposure to RXR-PNA or Tat-PNA, in contrast to the respective peptide conditions. In *E. coli*, this gene has recently been shown to encode a new transmembrane exporter, which mediates the export of, and thus conferring resistance to, 3-hydroxypropionate (106). Whether these systems play an active role in PPNA import or export will require further investigation. In this respect, our approach may pave the way to explore potential resistance mechanisms to PPNA exposure, as recently shown in the context of a carbapenem-resistant *E. coli* strain (10) and further illustrated by our finding that manipulation of the PhoP/Q system can affect sensitivity to PPNAs.

In conclusion, the RNA-seq-based analysis pioneered here provides valuable departure points for future research into the action mechanisms of PNA-based antimicrobials and the peptide-influenced physiological effect of PPNAs. Given the growing interest in selective antibiotics, this study provides a blueprint for future transcriptomic studies and could inform rational design strategies for eliminating or reprogramming many other bacterial species.

## DATA AVAILABILITY

RNA-seq data obtained in this study have been deposited with GEO under accession number GSE155764 (https://www.ncbi.nlm.nih.gov/geo/query/acc.cgi?acc=GSE155764).

## Supplementary Material

gkab242_Supplemental_Files
