# Supplementary material for: Global RNA profiles show target selectivity and physiological effects of peptide-delivered antisense antibiotics
Source: Nucleic Acids Res. 2021 Apr 13;49(8):4705–24. doi: 10.1093/nar/gkab242 (PMC8096218; doi:10.1093/nar/gkab242)
Supplement: gkab242_Supplemental_Files [file gkab242_supplemental_files.zip › SI_Popella et al., 2020_NAR_v08_LP.pdf]

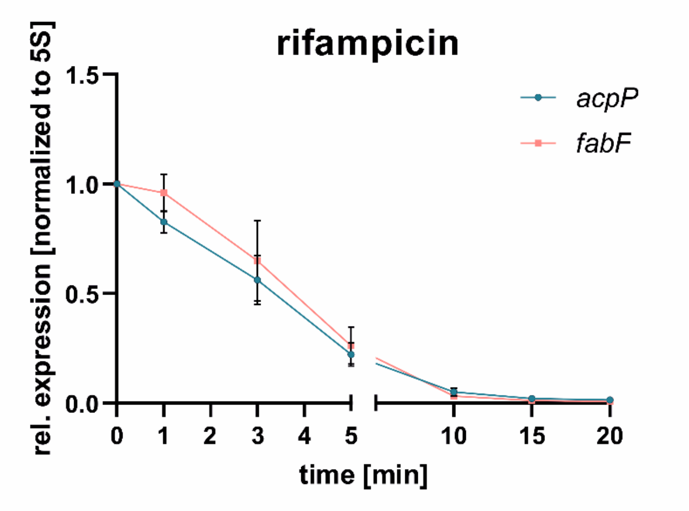

Fig. S1. Relative expression levels of *acpP* and *fabF* upon transcriptional arrest measured by RT-qPCR. Total RNA was extracted from 10^8 *Salmonella* cells at the indicated time points post treatment with 0.5 mg/ml rifampicin. Expression levels were normalized to the reference gene 5S. Data represent the average of three biological replicates, error bars indicate standard error of the mean.

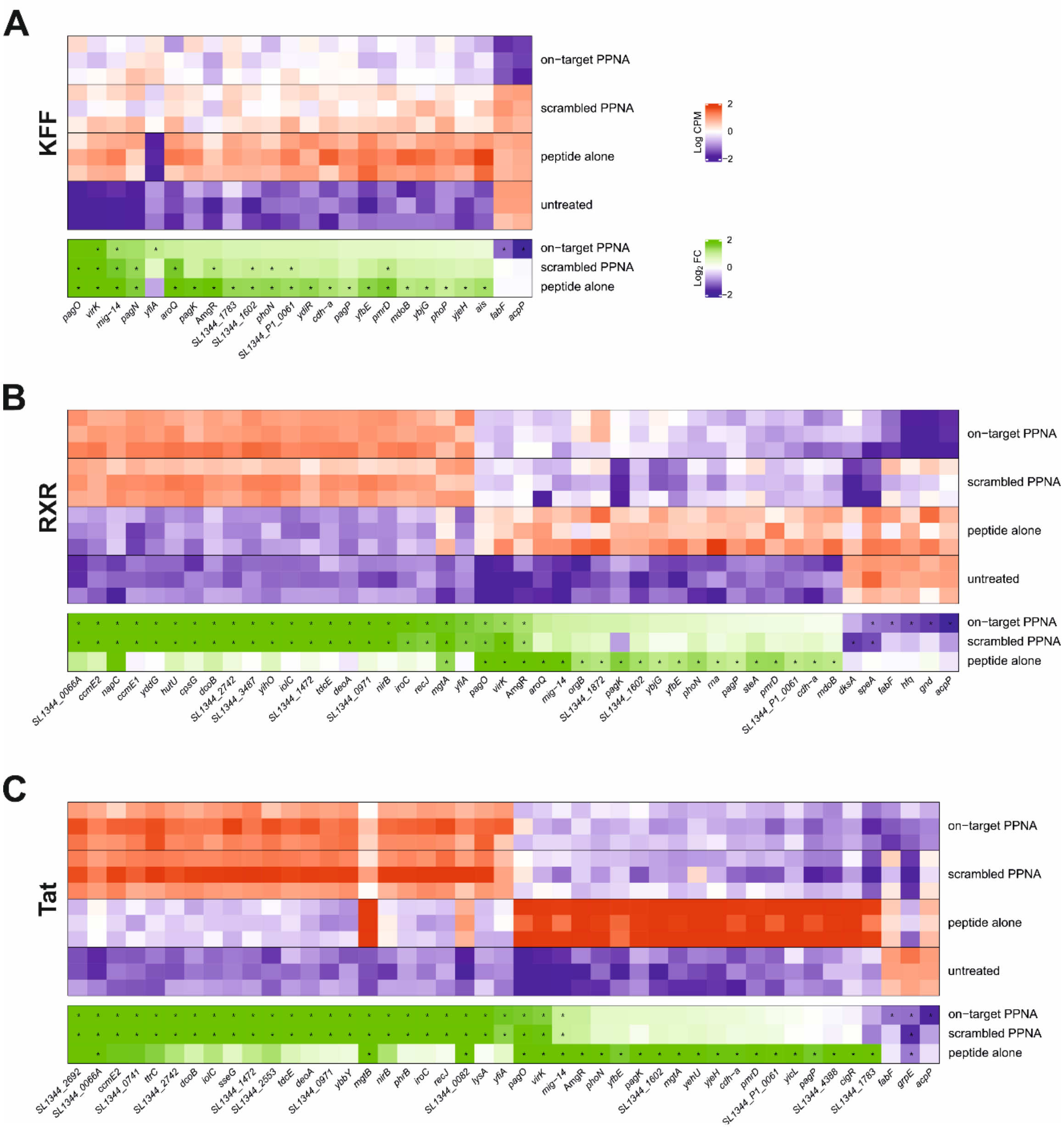

Fig. S2. Transcriptome profiles of *Salmonella* in response to treatment with peptide-PNA conjugates, as inferred from RNA-seq (experimental setup see Fig. 5A). (**A-C**) Calculated changes in *Salmonella* gene expression in response to different PPNA conjugates, as denoted on the left - (**A**) KFF, (**B**) RXR and (**C**) Tat. Each comparison includes triplicate RNA-seq samples for the on-target PPNA, a scrambled control PPNA, the peptide alone, and the untreated control. Upper panels show read counts per gene in column-centered log CPMs (counts per millions), while red and blue colors denote higher and lower expression, respectively. Lower panels show differential expression (log_2 fold change, FC) of genes in treated samples compared to the untreated controls. Green and purple colors denote positive and negative differences in gene expression, respectively. Only the top 20-regulated genes with an absolute FC value > 2 and p_{adj} < 0.001 (marked with asterisks) are shown in all samples, alongside the target gene *acpP* and its co-transcribed partner *fabF*.

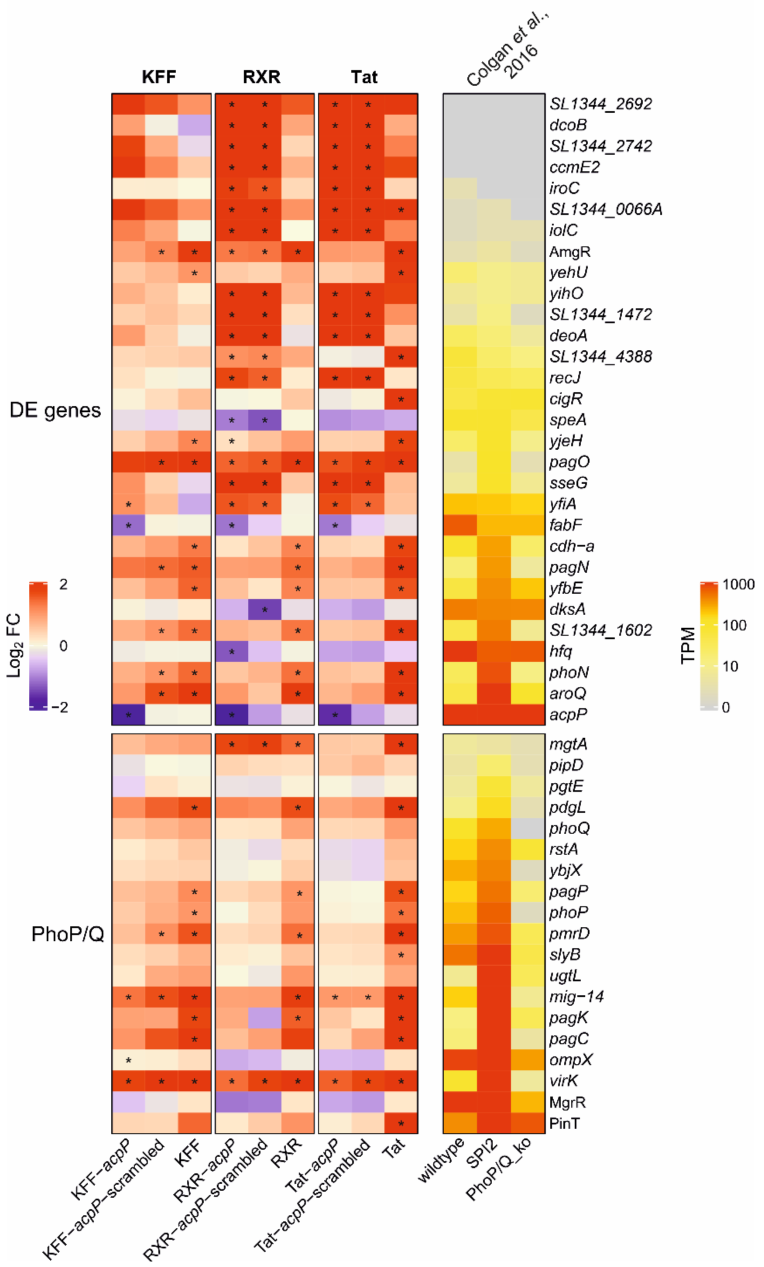

Fig. S3. Comparative analysis of gene expression data obtained in this study against datasets obtained in Colgan *et al.,* (34). Left panel shows $\log_2$ fold change (FC) of gene expression of the top 10-regulated transcripts in each of the nine indicated conditions (bottom left), while red and blue denote up- and down-regulation, respectively. Asterisks indicate significant regulation ($p_{adj} < 0.001$ and absolute FC > 2). Right panel: Transcripts per million (TPM) values of PhoP/Q-related genes in three conditions: Wildtype control, SPI2 and SPI2 with a PhoP/Q knockout strain. Wildtype - *Salmonella* wildtype grown under conditions without SPI-2 induction; SPI2 - *Salmonella* wild-type grown under conditions with SPI-2 induction; PhoP/Q_ko - *Salmonella* PhoP/Q-*knockout* strain in SPI2 inducing conditions.

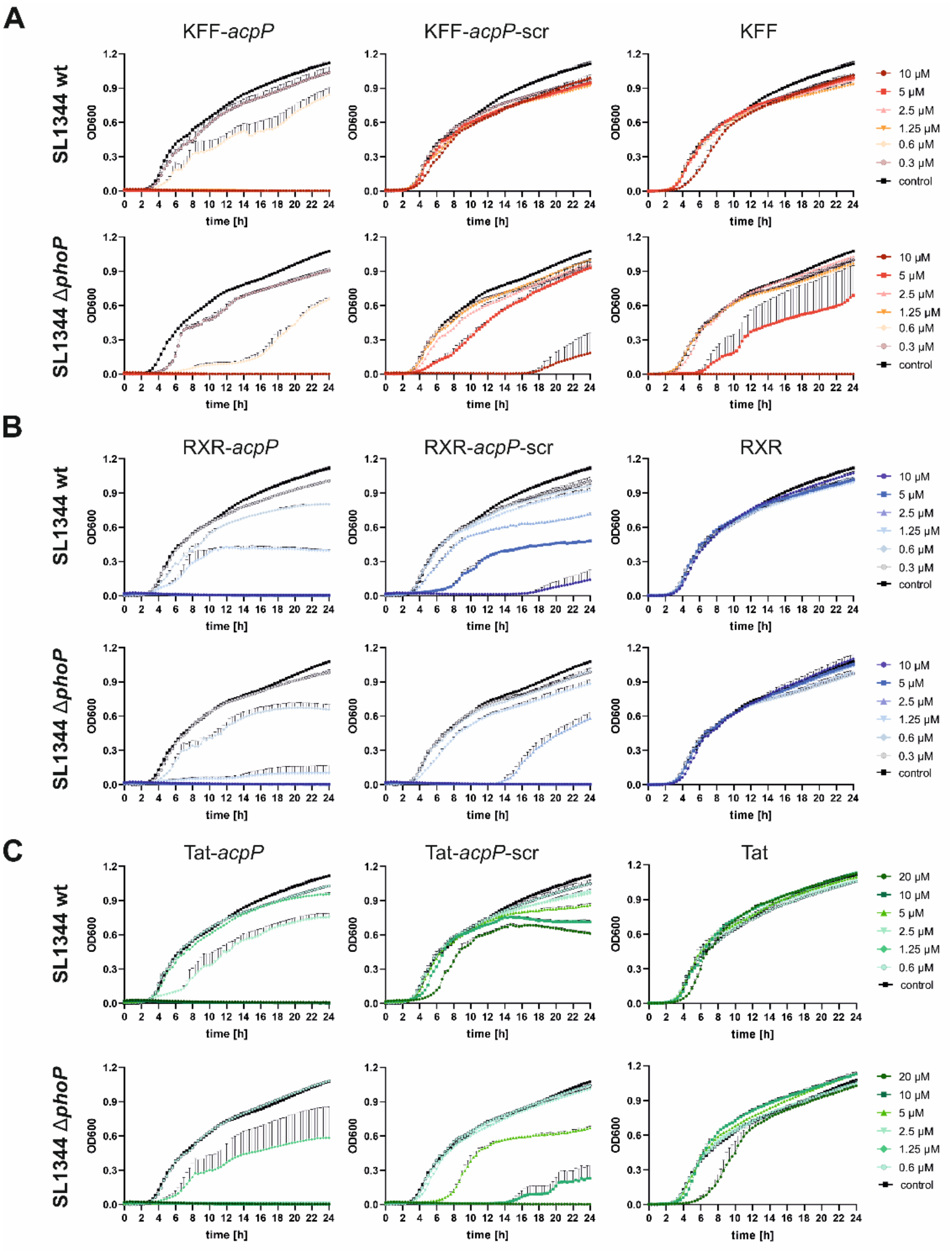

Fig. S4. Growth kinetics for MIC determination of 10^5 cfu/ml *Salmonella wt* (SL1344) and its isogenic Δ*phoP* mutant in the presence of varying concentrations of the indicated conjugates. (**A**) KFF-*acpP*, KFF-*acpP*-scrambled (scr) or KFF peptide in serial dilutions from 10 µM to 0.3 µM. (**B**) RXR-*acpP*, RXR-*acpP*-scrambled (scr) or RXR peptide in serial dilutions from 10 µM to 0.3 µM. (**C**) Tat-*acpP*, Tat-*acpP*-scrambled (scr) or Tat peptide in serial dilutions from 20 µM to 0.6 µM. Data represent the average of three biological replicates, error bars indicate standard error of the mean (SEM).

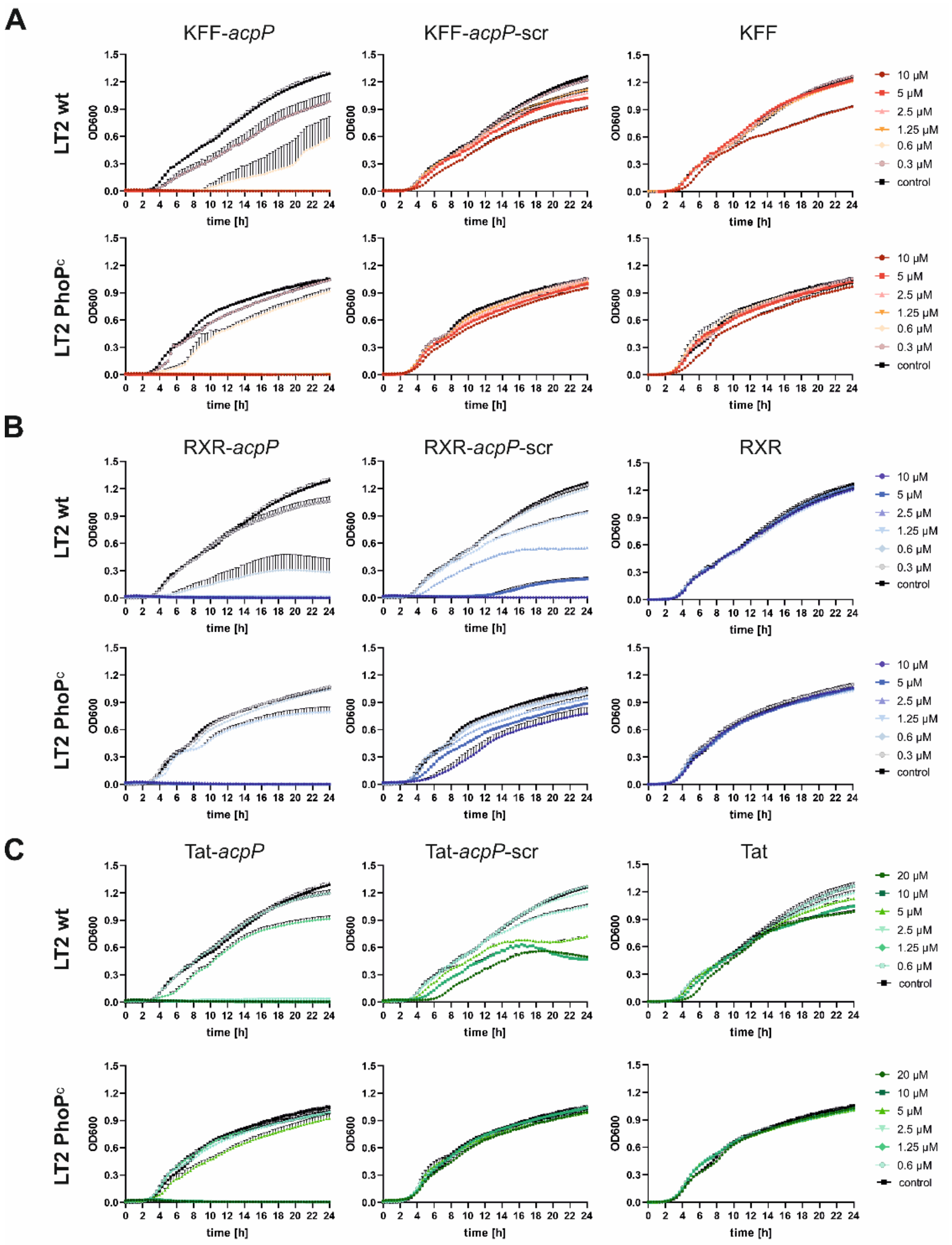

Fig. S5. Growth kinetics for MIC determination of 10^5 cfu/ml *Salmonella wt* (LT2) and its isogenic PhoP^C mutant in the presence of varying concentrations of the indicated conjugates. (**A**) KFF-*acpP*, KFF-*acpP*-scrambled (scr) or KFF peptide in serial dilutions from 10 µM to 0.3 µM. (**B**) RXR-*acpP*, RXR-*acpP*-scrambled (scr) or RXR peptide in serial dilutions from 10 µM to 0.3 µM. (**C**) Tat-*acpP*, Tat-*acpP*-scrambled (scr) or Tat peptide in serial dilutions from 20 µM to 0.6 µM. Data represent the average of three biological replicates, error bars indicate standard error of the mean (SEM).

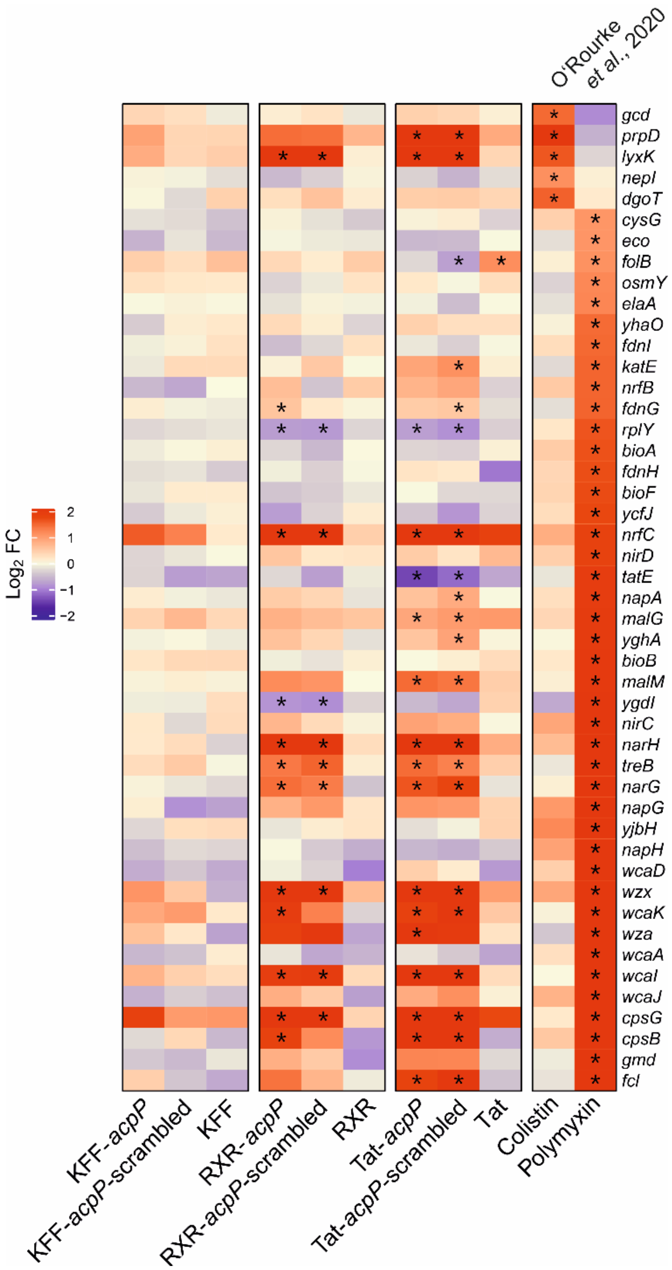

Fig. S6. Comparison of gene expression profiles of *Salmonella* obtained in this study in response to PPNA, PPNA-scrambled or peptide alone treatment with the published gene expression profiles of *E.coli* in response to colistin and polymyxin B treatment (35). Asterisks indicate significant regulation (p< 0.01 and absolute FC > 2). Heatmap color code is depicted at the left and indicates $\log_2$ fold changes.

Table S1. Purity and quality of the herein used PNA, PPNA and peptide constructs.

Name	Purity [%]	Calculated molecular mass [g/mol]	Detected molecular mass [g/mol]
KFF-*acpP*	99.931	4031.84	4031.811
KFF-*acpP*-scr	99.884	4031.84	4031.893
[a]KFF	> 98 %	1455.78	1455.81
RXR-*acpP*	99.552	4522.32	4522.717
RXR-*acpP*-scr	99.455	4522.32	4522.565
[a]RXR	> 98 %	1875.33	1875.36
Tat-*acpP*	99.932	4434.18	4434.364
Tat-*acpP*-scr	99.751	4434.18	4434.417
[a]Tat	> 98 %	1858.21	1858.25
ANT-*acpP*	99.651	4864.33	4863.975
ANT-*acpP*-scr	99.795	4864.33	4864.942
ANT	99.222	2245.29	2245.37
SAP-*acpP*	99.963	4615.29	4615.179
SAP-*acpP*-scr	99.561	4615.29	4615.865
SAP	98.201	1996.25	1996.219
SAPE-*acpP*	99.947	4534.11	4534.248
SAPE-*acpP*-scr	99.549	4534.11	4534.575
SAPE	99.841	1915.07	1915.141
acpP	100	2637.05	2636.868

[a] N-acetylated

Table S2. DNA oligonucleotides used for RT-qPCR or northern blot probe generation.

**Oligo (RT-qPCR)**	**Internal number**	**Sequence (5‘-3‘)**
acpP forward	JVO-16938	TGCTTCTTTCGTTTGAAGACC
acpP reverse	JVO-16939	TTTTCTCAGCTTCTTCGTCC
fabF forward	JVO-17745	TGACGGAAGAGAACGCAAG
fabF reverse	JVO-17746	ACAGGCAGTCGCAATAGAG
5S forward	JVO-16373	ACTAGCGCGGTGGTCCC
5S reverse	JVO-16374	GCAGTTCCCTACTCTCGATG
**Oligo (DIG-labeled NB probe)**	**Internal number**	**Sequence (5‘-3‘)**
acpP forward	JVO-18305	*GTTTTTTATGCAT*ACACTACGAAAACCATCGCGAAA
acpP reverse	JVO-19053	CTAATACGACTCACTATAGGGAGAGCTCAACGGTGTCAAGAGAATC
5S forward	JVO-16373	ACTAGCGCGGTGGTCCC
5S reverse	JVO-19055	CTAATACGACTCACTATAGGGAGAGCAGTTCCCTACTCTCGCATG
**Oligo (^{32}P-labeled NB probe)**	**Internal number**	**Sequence (5‘-3‘)**
SdhX	JVO-16831	CCGTAAAGGTGGCCAACCATGTCG
SroC	JVO-2907	GAAGATTGTTGCCCGGCGATTTG
5S	JVO-0322	CTACGGCGTTTCACTTCTGAGTTC

* NB: northern blot, DIG: digoxigenin, italic nucleotides denote sequence-unrelated nucleotides, underlined nucleotides denote T7 promoter sequence.

Dataset S1 (separate file). This dataset contains (i) a list of all raw counts for each experimental condition, (ii) the comparison of differential expression data for all identified transcripts in our RNA-Seq dataset involving three biological replicates, and (iii) the complete dataset for KEGG pathway analysis.
